# Proteomic Associations of N-terminal (NT)-pro hormone BNP (NT-proBNP) in Heart Failure with Preserved Ejection Fraction (HFpEF)

**DOI:** 10.1161/CIRCHEARTFAILURE.123.011146

**Published:** 2024-02-01

**Authors:** Joe David Azzo, Marie-Joe Dib, Loukas Zagkos, Lei Zhao, Zhaoqing Wang, Ching-Pin Chang, Christina Ebert, Oday Salman, Sushrima Gan, Payman Zamani, Jordana B. Cohen, Vanessa van Empel, A. Mark Richards, Ali Javaheri, Douglas L. Mann, Ernst Rietzschel, Peter Schafer, Dietmar A. Seiffert, Dipender Gill, Stephen Burgess, Francisco Ramirez-Valle, David A. Gordon, Thomas P. Cappola, Julio A. Chirinos

**Affiliations:** 1 University of Pennsylvania Perelman School of Medicine, Philadelphia, PA; 2 Division of Cardiovascular Medicine, Hospital of the University of Pennsylvania, Philadelphia PA; 3 Department of Epidemiology and Biostatistics, School of Public Health, Imperial College London, UK; 4 Bristol-Myers Squibb Company, Lawrenceville, NJ; 5 Renal-Electrolyte and Hypertension Division, Perelman School of Medicine, University of Pennsylvania, Philadelphia PA; 6 Department of Cardiology, Maastricht University Medical Center, Maastricht, The Netherlands; 7 Cardiovascular Research Institute, National University of Singapore, Singapore; 8 Christchurch Heart Institute, University of Otago, Christchurch, New Zealand; 9 Washington University School of Medicine, St. Louis, MO; 10 John J. Cochran Veterans Hospital, St. Louis, MO; 11 Department of Cardiovascular Diseases, Ghent University Hospital, Ghent, Belgium; 12 Department of Public Health and Primary Care, University of Cambridge, Cambridge, UK

**Keywords:** NT-proBNP, HFpEF, clinical trials, proteomics, canonical pathways

## Abstract

**Background:**

N-terminal-pro natriuretic peptide (NT-proBNP) levels are variably elevated in heart failure with preserved ejection fraction (HFpEF), even in the presence of increased left ventricular filling pressures. NT-proBNP levels are prognostic in HFpEF, and have been used as an inclusion criterion for several recent randomized clinical trials. However, the underlying biologic differences between HFpEF participants with high and low NT-proBNP levels remain to be fully understood.

**Methods:**

We measured 4,928 proteins using an aptamer-based proteomic assay (SOMAScan®) in available plasma samples from 2 cohorts: (1) Participants with HFpEF enrolled in the Penn Heart Failure Study (PHFS, *n*=253); (2) TOPCAT trial participants in the Americas (*n*=218). We assessed the relationship between SOMAScan®-derived plasma NT-proBNP and levels of other proteins available in the SOMAScan® assay version 4 using robust linear regression, with correction for multiple comparisons, followed by pathway analysis.

**Results:**

NT-proBNP levels exhibited prominent proteome-wide associations in PHFS and TOPCAT cohorts. Proteins most strongly associated with NT-proBNP in both cohorts included sushi, von Willebrand factor type-A, EGF and pentraxin domain containing-1 (SVEP1; β_TOPCAT_=0.539; *P*<0.0001; β_PHFS_=0.516; *P*<0.0001), and angiopoietin-2 (ANGPT2; β_TOPCAT_=0.571; *P*<0.0001; β_PHFS_=0.459; *P*<0.0001). Canonical pathway analysis demonstrated consistent associations with multiple pathways related to fibrosis and inflammation. These included hepatic fibrosis and inhibition of matrix metalloproteases. Analyses using cut-points corresponding to estimated quantitative concentrations of 360 pg/ml (and 480 pg/ml in atrial fibrillation) revealed similar proteomic associations.

**Conclusions:**

Circulating NT-proBNP levels exhibit prominent proteomic associations in HFpEF. Our findings suggest that higher NT-proBNP levels in HFpEF are a marker of fibrosis and inflammation. These findings will aid the interpretation of NT-proBNP levels in HFpEF and may guide the selection of participants in future HFpEF clinical trials.

## Abbreviations

TOPCAT: Treatment of Preserved Cardiac Function Heart Failure with an Aldosterone Antagonist
PHFS: Penn Heart Failure Study
SVEP1: sushi, von Willebrand factor type A, EGF and pentraxin domain containing 1
PKG1: cGMP-dependent protein kinase 1
ANGPT2: angiopoietin 2
PXDN: peroxidasin homolog

## Introduction

Heart failure (HF) with preserved ejection fraction (HFpEF) is a prevalent condition, and is a leading cause of morbidity, poor quality of life and mortality worldwide.^1^ This common condition has complex pathophysiology.^2^ Natriuretic peptides (NP) are extensively studied and widely used biomarkers for HF management in both HF with reduced ejection fraction (HFrEF) and HFpEF. B-type natriuretic peptide (BNP) and N-terminal pro-BNP (NT-proBNP) are the preferred biomarkers for guiding HF management. NT-proBNP specifically is the most widely utilized NP biomarker, due to its longer circulating half-life and higher plasma concentration.^3^

In contrast to HFrEF, NT-proBNP levels are not elevated in a significant proportion of patients with HFpEF, even in the presence of increased left ventricular filling pressures at rest.^4^ This is may be due to several factors, including the presence of non-dilated left ventricles with relatively thick walls, limiting the increase in wall stress associated with high LV pressures. In addition, comorbidities that are prevalent in HFpEF, including obesity, atrial fibrillation and renal disease,^5^ may confound the association between NT-proBNP and HFpEF, thereby mitigating its diagnostic and prognostic value. Despite these potential limitations, increased NT-proBNP levels have been used as a criterion for inclusion into several recent landmark randomized trials.^6–8^ Interestingly, some available data suggests that lower NT-proBNP levels may identify individuals who particularly responsive to angiotensin II-receptor blockade^9^ and/or spironolactone therapy.^10^ Therefore, the use of NT-proBNP for inclusion in HFpEF trials^11^ may select participants with specific biologic phenotypes. Moreover, the underlying biologic differences between HFpEF patients with high and low NT-proBNP levels are not well understood.

In this study, we aimed to assess underlying biologic differences between HFpEF participants with high and low NT-proBNP levels leveraging plasma proteomics data (~5,000 circulating proteins) from 2 HFpEF cohorts, coupled with pathway analysis.

## Methods

### Study population

The data supporting findings of this study may be made available for collaborative research upon the execution of appropriate data sharing agreements.

Data for this study were obtained from the Penn HF Study (PHFS, *n*=253) and the Treatment of Preserved Cardiac Function Heart Failure with an Aldosterone Antagonist Trial (TOPCAT, *n*=218). The PHFS is a prospective cohort study of HF patients recruited at the University of Pennsylvania (Philadelphia, PA), Case Western Reserve University (Cleveland, OH), and the University of Wisconsin (Madison, WI) between 2003-2011. Patients with clinical diagnosis of HF as determined by a HF specialist were enrolled. At the time of enrolment, standardized questionnaires were administered to participants and their physicians to obtain detailed clinical data. Participants with expected mortality of 6 months or less from a non-cardiac condition, as judged by their treating physician, mechanical circulatory support, or inability to provide informed consent were excluded. The PHFS cohort included 253 participants with HFpEF who had available plasma samples for proteomic quantification, which were included in this analysis.

TOPCAT is a randomized, double-blinded, international trial of spironolactone therapy versus placebo that enrolled 3,445 patients with HFpEF from 2006 to 2012. Key inclusion criteria were age 50 years or older, left ventricular ejection fraction ≥45%, at least one sign and one symptom of HF, and at least one hospital admission for HF within 12 months prior to study entry or a circulating BNP level ≥100 pg/ml or NT-proBNP ≥360 pg/ml within 60 days prior to study entry. Clinical data from the parent trial used for this study are available to researchers through the National Institutes of Health BioLINCC. Frozen blood plasma samples were available from the National Institutes of Health for proteomic quantification in 218 participants. This constituted a subset of 3,445 trial participants (6.3%), all of whom were enrolled in United States and Canada.

### IRB approval

In PHFS, an institutional review board from each participating center approved the PHFS protocol. Informed consent was also provided by the study participants. TOPCAT was conducted with the approval of local institutional review boards. Informed consent was also provided by the study participants.

### Plasma protein quantification

All plasma samples were analyzed using the SOMAScan® assay version 4, (SomaLogics Inc., Boulder, Colorado), which is a multiplexed, modified aptamer-based binding-assay. The SOMAScan® assay uses slow-off-rate modified aptamer (SOMAmer) reagents, which are chemically modified nucleotides, to bind and quantify target proteins in relative fluorescent units directly proportional to the amount of target protein in the sample. The SOMAScan® assay included 4,996 modified aptamer reagents to 4,928 unique protein targets.

### Statistical Analysis

Participant characteristics were summarized using mean for continuous variables with normal distribution and median (interquartile range, IQR) for continuous variables with skewed distribution. Categorical variables are expressed as counts (percentages). ANOVA was used to compare normally distributed continuous variables, whereas the Kruskal-Wallis test was used for non-normally distributed variables, and the χ^2^ or Fisher exact test, as appropriate, was used for categorical data. We performed stratified analysis of the study populations and compared key characteristics across tertiles of SOMAScan®-derived NT-proBNP.

**Figure 1:**
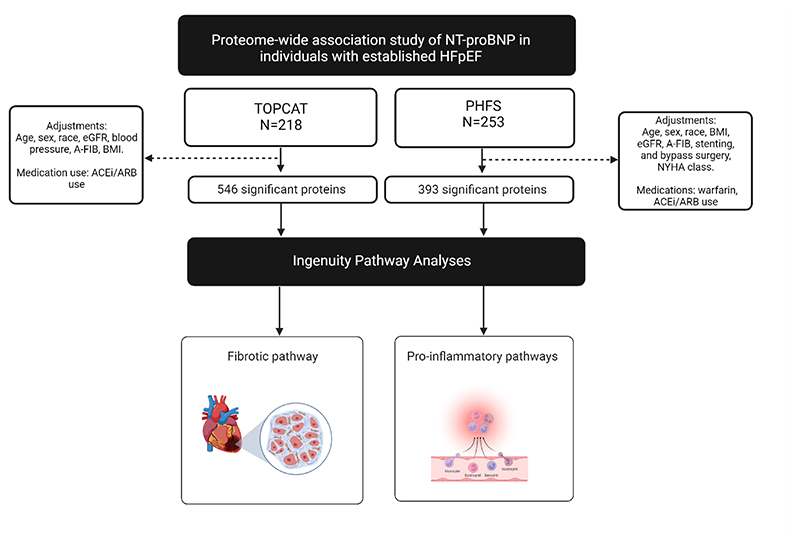
Proteome-wide association study of 4,996 plasma proteins on NT-proBNP levels. Robust linear regressions were performed on the Treatment of Preserved Cardiac Function Heart Failure with an Aldosterone Antagonist Trial (TOPCAT) and the Penn Heart Failure Study (PHFS) cohorts to identify proteins significantly associated with NT-proBNP.

A general representation of our inferential analytic approach is shown in Figure 1. We assessed the relationship between SOMAScan®-derived plasma NT-proBNP and levels of other proteins available in the SOMAScan® assay version 4 using robust linear regression which employs the iteratively reweighted least squares (IRLS) method to assign a weight to each data point, reducing outlier effects on regression models. We used the fitlm Matlab function using bisquare weight function with the default tuning constant (4.685). All regression coefficients (β) are standardized (expressed per SD increase) to facilitate comparisons between different plasma proteins. We corrected the alpha level for multiple comparisons using the number of principal components (PCs) underlying >95% of the variability of all measured proteins, as previously described.^12–15^ A total of 182 PCs in TOPCAT, and 209 PCs in PHFS, of which the eigenvalues cumulatively explained >95% of the variation observed in the measured proteins, were found. We also corrected the alpha level for multiple comparisons using the Benjamini & Hochberg procedure for estimating the false discovery rate (FDR) with a q-value not greater than 5% considered significant.^16^ In addition to univariable analyses, we built multivariable models for each protein, which adjusted for sex, race, eGFR, and any characteristics that varied significantly across tertiles of SOMAScan®-derived NT-proBNP in each cohort.

Statistical significance was defined as multiplicity corrected *P*-value <0.05. All probability values presented are 2-tailed. Analyses were performed using MATLAB statistics and machine learning toolbox (Matlab 2021b, the Mathworks; Natwick, MA) and R Statistical Software v3.5.2 (Foundation for Statistical Computing, Vienna, Austria).

### Pathway Analyses

Proteome-wide associations that were identified when adjusting for clinical factors related to NT-proBNP were utilized to perform pathway analyses, using Ingenuity Pathway Analysis software (Qiagen; Hilden, Germany). Proteins were identified according to their UniProt identifier annotation. The totality of proteins included in SOMAScan® assay was used as the reference set and both direct and indirect experimentally confirmed relationships from all species were included. The Core analysis module in Ingenuity Pathway Analysis was used to perform pathway analysis on the differentially expressed proteins. This analysis identifies specific canonical pathways in which the changes are highlighted. The analysis calculates a *P*-value (Fisher exact test and right tailed), quantifying the overlap, and a Z score, quantifying the likelihood and direction (up or downregulated), between the plasma proteomics pattern and known canonical pathways.

### Sensitivity analysis

Given that differences between participants above and below specific cut-points may be more directly relevant for issues related to inclusion into randomized clinical trials in HFpEF, we performed a sensitivity analysis comparing participants with NT-proBNP levels above and below currently recommended cut-points for inclusion.^11^ We utilized a cut-point of ≥360 pg/ml for participants without atrial fibrillation, with a 30% increase in the cut-point (≥480 pg/ml) for those with atrial fibrillation.^11^ Given that SOMAScan®-derived NT-proBNP levels do not provide absolute concentrations equivalent to those assessed with other available tests, we performed a linear regression analysis of SOMAScan®-derived NT-proBNP levels and levels measured with a quantitative independent assay (ARCHITECT immunoassays, Abbott Laboratories, Abbott Park, IL)^17^ among 68 PHFS participants (Figure S1). We then derived the appropriate SOMAScan®-derived NT-proBNP level cut-points corresponding to 360 and 480 pg/ml, as above in all HFpEF participants with available data. Finally, we computed standardized (z-score) differences in all other proteins in the SOMAScan® between the groups using ANOVA, correcting the alpha level for multiple comparisons using the number of principal components underlying >95% of the variability of all measured proteins.

## Results

### Baseline clinical characteristics

The general characteristics of study participants from the Americas in the TOPCAT trial with and without available proteomics data are shown in Table S1.

Baseline characteristics of PHFS and TOPCAT participants included in our study, stratified by tertiles of NT-proBNP, are shown in Tables 1-2, respectively. In PHFS, participants in higher tertiles of NT-proBNP were more likely to be older, exhibited a lower BMI, lower estimated glomerular filtration rate (eGFR), more advanced NYHA functional class, higher prevalence of atrial fibrillation, history of coronary revascularization, warfarin use and lower prevalence of ACE inhibitor and ARB use (Table 1).

In TOPCAT, participants in higher NT-proBNP tertiles were more likely to be older, and exhibited significantly lower body mass index (BMI), lower insulin use, and were more likely to have atrial fibrillation (Table 2). Participants in the middle tertile were less likely to have hypertension and use angiotensin-converting enzyme (ACE) inhibitors and angiotensin receptor blockers (ARB), compared to the other 2 tertiles.

### Correlation to other proteins and biologic pathways in PHFS

In PHFS, we found 1,006 proteins to be significantly associated with the plasma levels of NT-proBNP in univariable analyses. A volcano plot showing the relationship between NT-proBNP and other plasma protein levels is shown in Figure S2. Beta estimates and P-values for the top 50 proteins are shown in Table S2.

The top 5 proteins positively associated with plasma levels of NT-proBNP were scavenger receptor class F member 2 (SCARF2, β=0.711, *P*<0.0001), sushi, von Willebrand factor type A, EGF and pentraxin domain containing 1 (SVEP1, β=0.666, *P*<0.0001), EGF-containing Fibulin-like extracellular matrix protein 1 (EFEMP1, β=0.668, *P*<0.0001), insulin-like growth factor binding protein 2 (IGFBP2, β=0.654, *P*<0.0001), latent-transforming growth factor beta-binding protein 4 (LTBP4, β=0.634, *P*<0.0001). The top 5 proteins with a negative correlation with plasma levels of NT-proBNP were Cadherin-3 (CDH3, β=-0.655, *P*<0.0001), Growth hormone receptor (GHR, β=-0.595, *P*<0.0001), palmitoleoyl-protein carboxylesterase (NOTUM, β=-0.582, *P*<0.0001), Ficolin-3 (FCN3, β=-0.53, *P*<0.0001) and Heparan sulfate glucosamine 3-O-sulfotransferase 5 (HS3ST5, β=-0.522, *P*<0.0001).

**Figure 2:**
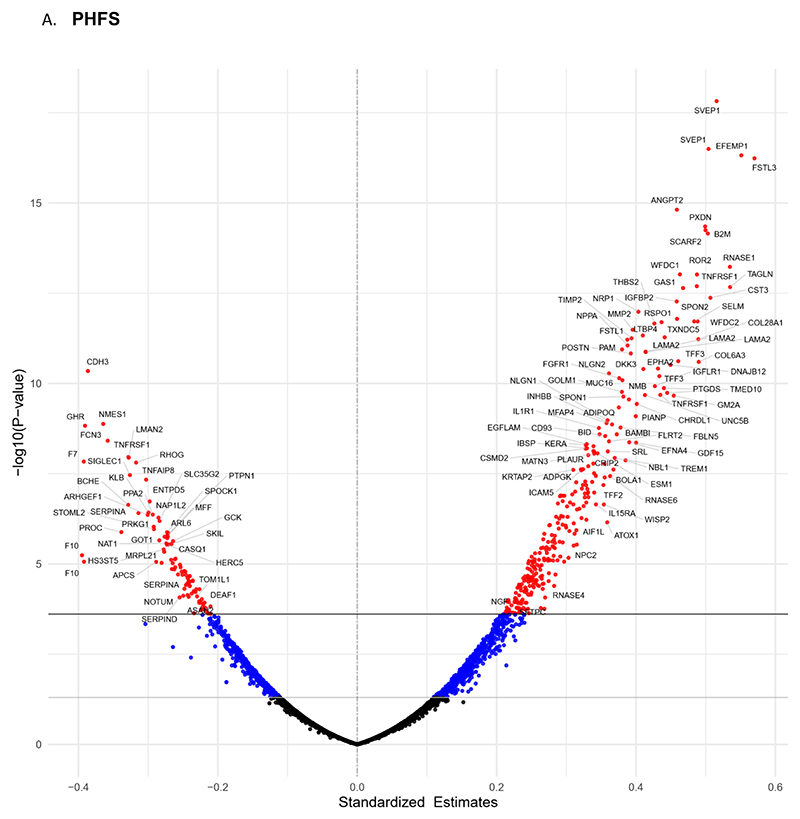

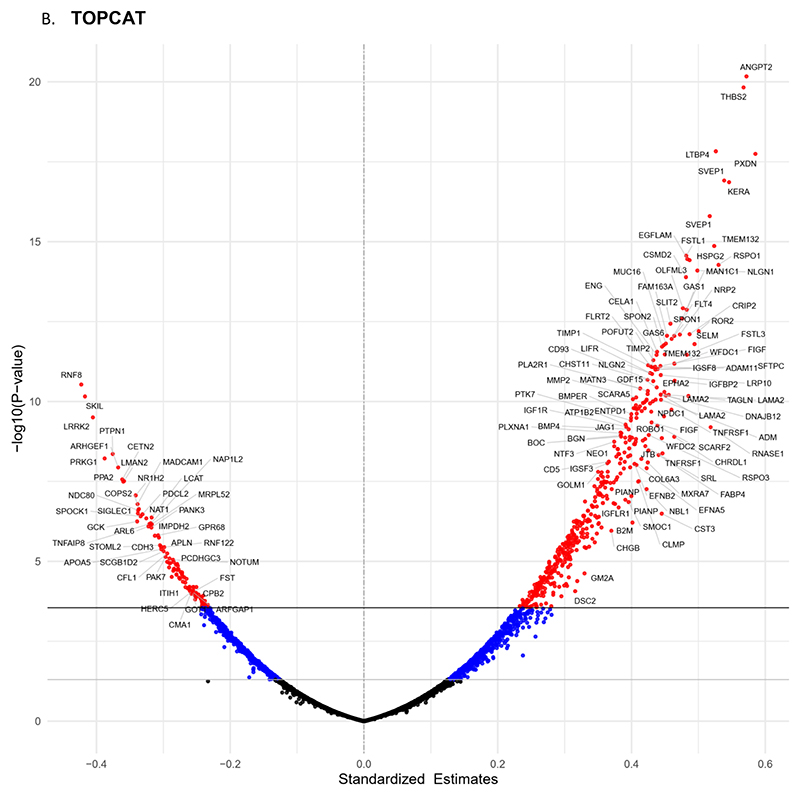
Volcano plot demonstrating significant associations between all plasma proteins with plasma levels of N-terminal-pro BNP (NT-proBNP) after adjusting for covariates measured in (A) the Penn Heart Failure Study (PHFS, n=253) and (B) the Treatment of Preserved Cardiac Function Heart Failure with an Aldosterone Antagonist Trial (TOPCAT, n=218). The plots show standardized beta estimates against the log-10 p value. The nominal and alpha-corrected significance levels are represented by solid lines on the y-axis.

Plasma levels of NT-pro BNP were found to be significantly associated with 393 proteins, after adjusting for sex, race, eGFR and additional covariates found to be significantly different across NT-proBNP tertiles (age, history of stenting, bypass surgery, atrial fibrillation, warfarin use, ACEI/ARB use and BMI, NYHA class; Figure 2A). Beta estimates and P-values for the top 50 proteins are shows in Table S3. The top 5 proteins with a positive correlation to plasma levels of NT-proBNP included Follistatin-related protein 3 (FSTL3, β=0.57, *P*<0.0001), SVEP1 (β=0.516, *P*<0.0001), EFEMP1 (β=0.551, *P*<0.0001), peroxidasin homolog (PXDN, β=0.499, *P*<0.0001) and ANGPT2 (β=0.459, *P*<0.0001). The top 5 proteins with a negative correlation to plasma levels of NT-pro BNP included CDH3 (β=-0.386, *P*<0.0001), Coagulation factor VII (F7, β= -0.392, *P*<0.0001), GHR (β=-0.39, *P*<0.0001), Coagulation factor X (F10, β=-0.394, *P*<0.0001) and FCN3 (β=-0.358, *P*<0.0001).

**Figure 3:**
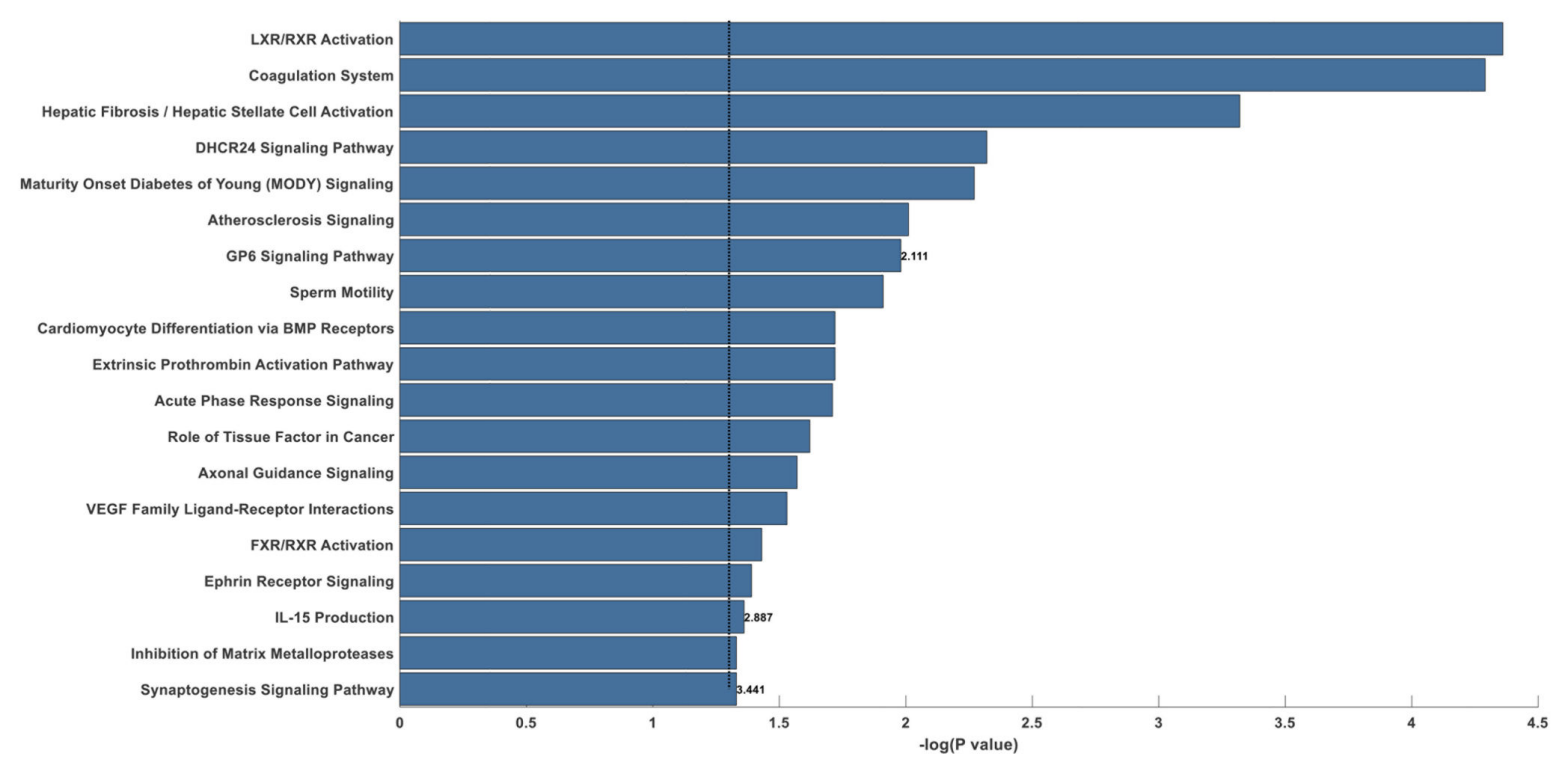

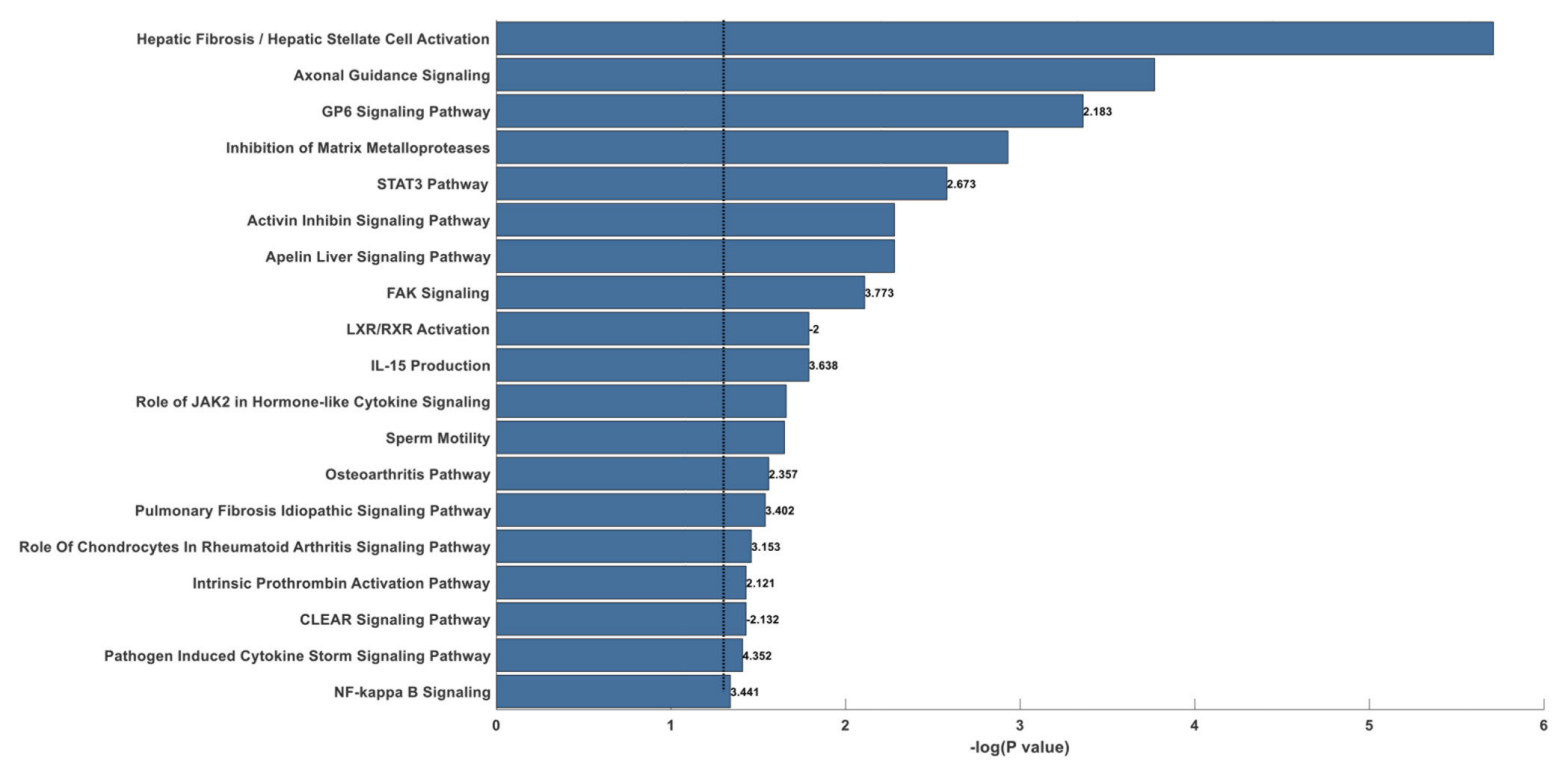
Canonical pathway analysis of proteins observed to be significantly associated with plasma levels of NT-proBNP levels in (A) the Penn Heart Failure Study (PHFS, n=253) and (B) the Treatment of Preserved Cardiac Function Heart Failure with an Aldosterone Antagonist Trial (TOPCAT, n=218). PCA corrected p-value of 0.05 threshold was used to determine significance. Numbers at the end of the bars indicate the Z-score corresponding to direction and strength of association.

The top canonical pathways significantly associated with NT-pro BNP in adjusted analyses in PHFS are shown **in** Figure 3A. The top 5 signaling pathways that were correlated with NT-proBNP included LXR/RXR activation pathway, hepatic fibrosis / hepatic stellate cell activation pathway, DHCR34 signaling pathway, the coagulation system pathway and maturity onset diabetes of young (MODY) signaling.

Sensitivity analyses using cut-points recommended as inclusion criteria into clinical trials, as opposed to continuous NT-proBNP levels as above, revealed 262 proteins associated with NT-proBNP levels above recommended cut-points, most of which (n=207) were concordant with linear regression results presented above (Figure S3). Similarly, canonical pathway analysis revealed several multiple concordant pathways (Figure S4).

### Correlation to other proteins and biologic pathways in TOPCAT

In the TOPCAT cohort, we found 1,946 proteins to be associated with NT-proBNP in univariable analyses. A volcano plot showing the relationship between NT-proBNP and other plasma protein levels is shown in Figure S5. Beta estimates and P-values for the top 50 proteins are shows in Table S4.

The top 5 proteins positively associated with plasma levels of NT-proBNP were thrombospondin-2 (THBS2, β=0.618, *P*<0.0001), angiopoietin-2 (ANGPT2, β=0.598, *P*<0.0001), SVEP1 (β=0.579, *P*<0.0001), keratocan (KERA, β=0.579, *P*<0.0001 and LTBP4 (β=0.586, *P<*0.0001). In addition, the top 5 proteins with negative correlation to plasma levels NT-proBNP were leucine-rich repeat serine/threonine-protein kinase 2 (LRRK2, β=-0.464, *P*<0.0001), ski-like protein (SKIL, β=-0.452, *P*<0.0001), E3 ubiquitin-protein ligase RNF8 (RNF8, β=-0.451, *P*<0.0001), mucosal vascular addressin cell adhesion molecule 1 (MAdCAM-1, β=-0.406, *P*<0.0001) and centrin-2 (CETN2, β=-0.405, *P*<0.0001).

The results of regression analyses between plasma levels of NT-proBNP and all other proteins in the SOMAScan®, adjusted for sex, race, eGFR and additional covariates found to be significantly different across NT-proBNP tertiles (age, hypertension, atrial fibrillation, BMI, insulin use, ACEi/ARBs use) are shown in Figure 2B. Plasma levels of NT-proBNP were found to be significantly associated with 546 proteins in these analyses. Beta estimates and P-values for the top 50 proteins are shows in Table S5. The top 5 proteins with a positive correlation to NT-pro BNP included ANGPT2 ((β=0.571, *P*<0.0001), THBS2 (β=0.567, *P*<0.0001), PXDN (β=0.585, *P*<0.0001), KERA (β=0.545, *P*<0.0001), and SVEP1 (β=0.539, *P*<0.0001). On the other hand, the top 5 proteins with negative correlation to NT-proBNP were, RNF8 (β=-0.422, *P*<0.0001), SKIL (β=-0.417, *P*<0.0001), cGMP-dependent protein kinase 1 (PKG1, β=-0.388, *P*<0.0001), LRRK2 (β=-0.405, *P*<0.0001) and Tyrosine-protein phosphatase non-receptor type 1 (PTPN1, β=-0.376, *P*<0.0001).

We utilized adjusted regression results for pathway analyses (Figure 3B). The top canonical pathways associated with NT-proBNP in TOPCAT were: hepatic fibrosis/stellate cell activation pathway, axonal guidance pathway, STAT 3 pathway, inhibition of matrix metalloproteases pathway and GP6 signaling pathway.

### Concordance Analysis between the 2 cohorts

**Figure 4:**
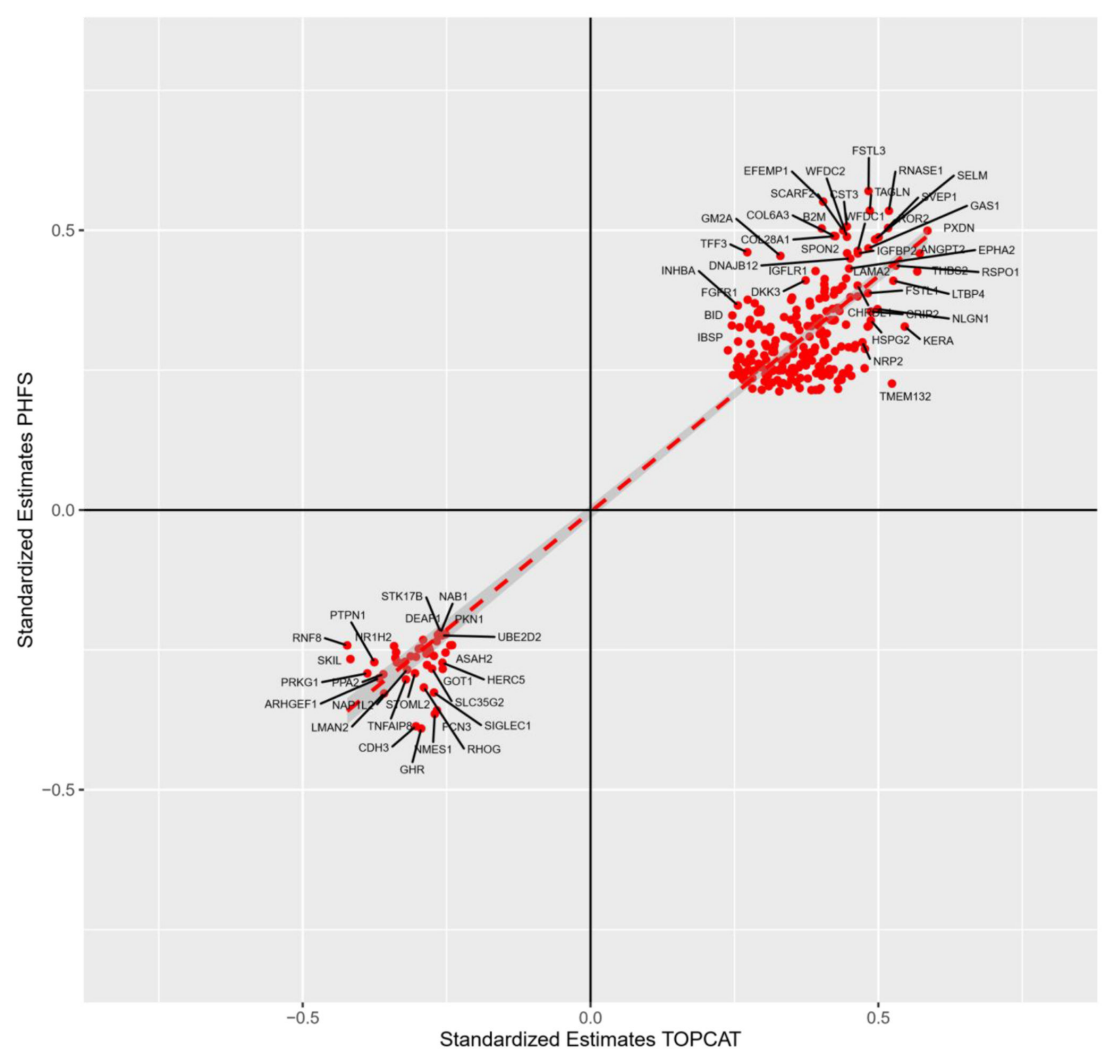
Concordance between standardized beta estimates for proteome-wide association analyses in PHFS and TOPCAT.

A concordance plot in which the proteins that were positively or negatively related to NT-proBNP in both cohorts is shown in Figure 4. We found 289 aptamers corresponding to 278 proteins that were concordantly and significantly associated with NT-proBNP in both cohorts. The top concordant proteins positively associated with NT-proBNP included SVEP-1, PXDN, ANGPT2, Receptor Tyrosine Kinase Like Orphan Receptor 2 (ROR2) and R-spondin-1 (RSOP1). The top concordant proteins negatively associated with NT-proBNP included CDH3, lectin mannose binding 2 (LMAN2), GHR, PRKG1 and FCN3.

### Sensitivity Analysis

We performed a sensitivity analysis comparing participants with NT-proBNP levels above and below currently recommended cut-points for inclusion (360 and 480 pg/ml). The results of the sensitivity analysis are shown on the online supplement (Table S6-S8, Figure S3, S4, S6).

## Discussion

To our knowledge, this is the first comprehensive proteomic analysis investigating the relationship between plasma NT-proBNP and other plasma protein levels in HFpEF. In 2 well-characterized, independent HFpEF cohorts, we identified novel associations between multiple plasma proteins and NT-proBNP, which indicate a relationship to pathways involved in ECM formation/fibrosis, inflammation, cellular regulation, and angiogenesis. Furthermore, we performed concordance analyses, adding confidence to our results. Our study advances our understanding of the clinical and biologic correlates of this widely used biomarker in HFpEF.

### Plasma proteins associated with NT-proBNP in HFpEF

In adjusted analyses, we identified 393 proteins significantly associated with NT-proBNP in the PHFS cohort, the vast majority of which (n=287) replicated in the TOPCAT cohort in both significance and directionality, adding confidence to the generalizability of our results. The top concordant plasma proteins associated with NT-proBNP included SVEP-1, PXDN, ANGPT2, and PRKG1. Across the two study groups, cellular pathways related to inflammation and fibrosis were significantly associated with NT-proBNP.

ANGPT2 was among the top proteins positively associated with NT-proBNP in both cohorts. Angiopoietins are growth factors involved in angiopoietin-tie-ligand-receptor system which plays an essential role in angiogenesis^18^ and in the maintenance of vascular integrity.^19^ More specifically, ANGPT2 destabilizes the resting endothelium and primes it to respond to external stimuli, facilitating the activity of inflammatory cytokines.^20^ ANGPT2 is weakly expressed in the resting endothelium, and its expression increases following endothelial activation.^21^ In the presence of VEGF (a protein which we also identified in this study as an NT-proBNP correlate), ANGPT2 promotes basal lamina remodeling, and new vessel growth.^22^ Animal studies have shown that overexpression of ANGPT2 reduces VEGF expression, and is consequently involved in impaired angiogenesis and worsened cardiac fibrosis.^23^ ANGPT2 inhibition in mice alleviated inflammation and cardiac hypoxia while improving post-ischemic cardiovascular remodeling.^24^ Similarly, human studies have shown that ANGPT2, tie-2 and VEGF levels were elevated in acute congestive HF when compared with controls.^25^

SVEP1, also called polydom, is a large extracellular matrix protein known to interact with integrin-α9β1, a matrix-binding integrin expressed in lymphatic system endothelial cells,^26^ which plays a role in cell adhesion and migration and promotes inflammatory responses.^27^ Another known ligand of integrin-α9β1 is tenascin-C (TNC), which has been shown to be associated with fibrosis and adverse outcomes in HFpEF.^28^ A possible role for SVEP1 in established HF can thereby be hypothesized to occur through its interaction with TNC and integrin, and their downstream signaling pathways leading to inflammation and fibrosis. Available data also support a role for SVEP1 in atherosclerosis^29,30^ although some evidence is conflicting.^31^ The mechanistic relationship between SVEP1 and NT-proBNP requires further study.

Plasma levels of PKG1 were negatively associated with NT-proBNP in both cohorts. PKG1 is a serine/threonine protein kinase that serves as an important mediator of the cGMP signaling pathway, is expressed in the cardiovascular system, and is a key mediator of the cellular effects of natriuretic peptides (NP) and nitric oxide (NO).^32^ Important biologic processes in which PKG is involved include tissue fibrosis, regulation of titin stiffness,^33^ and myocardial hypertrophy. Some groups have proposed that, as part of the pathogenesis of HFpEF, various comorbidities lead to a systemic inflammatory state that causes oxidative stress in the coronary microvascular endothelium, resulting in reduced PKG activity in cardiomyocytes, causing them to become fibrotic and hypertrophied.^2,34^ Interestingly, biopsy samples from HFpEF patients showed reduced PKG activity and cGMP concentrations.^34^ The negative relationship between PKG and NT-proBNP may result from more advanced HFpEF leading to greater natriuretic peptide release, although potential reverse causality or an extracardiac source of circulating PKG cannot be excluded. In addition, it should be noted that, although BNP is known to affect intracellular PKG1 activity, whether plasma levels of PKG1 are in any way representative of cellular PKG1 activation remains to be determined.

We found that PXDN (also known as vascular peroxidase 1, VPO1), was among the top proteins positively associated with NT-proBNP in both cohorts. VPO1 is highly expressed in the cardiovascular system,^35^ and can induce cardiomyocyte apoptosis,^36^ aggravate cardiac fibrosis in myocardial infarction^37^ in mice. However, little is known about the role of VPO1 in human HF, or its relationship to NT-proBNP. Further research is required to assess the basis for the relationship with NT-proBNP found in our study.

Atrial natriuretic peptide was not among the top significant proteins associated with NT-proBNP in our analyses of 2 independent HFpEF cohorts. Although previous studies have indicated that plasma concentrations of ANP and NT-proANP are strongly correlated to NT-proBNP levels,^38^ Reginauld et al. suggest that ANP and BNP may be differentially regulated in HF with the possible existence of a ANP-deficient subgroup among HF patients.^39^ The presence of an alternate mechanism of regulation between ANP and BNP is also supported by the increased susceptibility to degradation of ANP compared to BNP by neprilysin.^40,41^

### Pathway analyses

Using canonical pathway analyses, we identified several biological pathways associated with plasma NT-proBNP levels in HFpEF. Across both cohorts, inflammation and organ fibrosis were two prominent processes highlighted in the analysis.

Fibrosis is known to be an important process in HFpEF. We identified several proteins involved in tissue fibrosis (SVEP1, matrix metalloprotease 2, tissue inhibitor of metalloproteinases 1 and 2, PXDN), as significant correlates of plasma NT-proBNP in both cohorts. Accordingly, various pathways related to fibrosis were associated with NT-proBNP in canonical pathway analyses. Importantly, BNP has been reported to have anti-fibrotic activity. For instance, BNP reduced collagen synthesis in adult canine cardiac fibroblasts, and regulated levels of various MMPs and TIMPs.^42^ In addition, an increase in focal fibrotic lesions in the ventricles and pro-fibrotic factors were also seen in BNP-deficient mice.^43^

We also identified several proteins involved in inflammation (TNFRSF1A, TNFRSF1B, TNFRSF11, IL1R1) as significant correlates of plasma NT-proBNP. Several inflammatory pathways were associated with NT-proBNP in canonical pathway analyses in both cohorts. Previous evidence links natriuretic peptide levels to inflammation in humans. Fish-Trotter and Ferguson *el al* reported an independent relationship between plasma NT-proBNP and circulating IL-6 in the Multi-Ethnic Study of Atherosclerosis, as well as relationship between a number of inflammatory conditions and NT-proBNP in a large sample of hospitalized patients.^44^ Moreover, they found that administration of lipopolysaccharide (LPS) to healthy volunteers resulted in a marked increase in circulating NT-proBNP.^44^ A similar relationship between IL-6 and NT-proBNP was reported in the British Regional Heart Study (a general population sample), in which the relationship between IL-6 and incident HF was markedly attenuated after adjustment for NT-proBNP.^45^

Finally, in sensitivity analyses using cut-points recommended as inclusion criteria into clinical trials, as opposed to continuous NT-proBNP levels, we identified largely similar proteins and pathways associated with high NT-proBNP. Therefore, by using NT-proBNP levels as an inclusion criterion in clinical trials with currently recommended thresholds, investigators may select HFpEF participants with particularly biologic profiles, which might have implications on the outcomes of trials with specific agents. This may be useful in the design of future trials, particularly with agents that may act on pathways that are differentially expressed in patients with high vs. low NT-proBNP levels.

### Strengths and limitations

The findings of our study should be interpreted in context of its strengths and limitations. Given the continuous nature of our measurements, our sample sizes were sufficient for identifying a plethora of novel proteins associated with NT-proBNP, an important clinical biomarker in HFpEF. Our study combined participants with established HFpEF from 2 different cohorts which allowed us to reproduce and validate our results, a broad proteomics platform of ~5000 plasma protein measurements, pathway analyses and concordance analysis (which add confidence and support the generalizability of our observational findings). On the other hand, our study also has significant limitations. Since we based our analyses on specific HFpEF cohorts, inclusion into these studies may carry some selection bias. In particular, the enrollment of a subset of participants in TOPCAT trial was based on levels of NT-proBNP, which could have introduced selection bias. Our measurements of NT-proBNP were made with SOMAScan®, rather than with traditional clinically utilized methods. Our populations were composed predominantly of Caucasians (with some significant representation of African-American in PHFS), and may not be generalizable to other race groups or other geographic locations. Furthermore, there were significant differences between the baseline characteristics of our participants with proteomics data in TOPCAT and other participants in this trial, which may also limit the generalizability of our results. The cohorts included in this study are not contemporary, and plasma samples were frozen for 1-2 decades prior to proteomics analyses, which may have affected our results. We did not assess measures of cardiac structure and function, because they were available in a small subset of the study populations. We also note that we did not aim to assess the clinical value of additional biomarkers in this context, but rather establish the proteomic correlates (and in turn, infer biologic correlates) of plasma levels of NT-proBNP, an increasingly utilized biomarker for selection into clinical trials. These correlates may or may not be causal, may be subject to unmeasured confounding, and therefore should not be overinterpreted. We did not assess whether certain factors (such as obesity, sex, age or eGFR) may modify the association between NT-proBNP and the rest of the plasma proteome, which should be assessed in future studies. Finally, we were not able to assess causality. It should be noted that NT-proBNP is in itself not biologically active (but rather a down-product of BNP secretion) and is subject to variable degradation by neprilysin and NP-receptor-mediated clearance mechanisms. Therefore, associations reported herein should not be assumed to be causal. Despite this consideration, these associations, whether causal or not, are relevant to our understanding of biologic correlates of this highly clinically-relevant phenotype, and by extension, of important differences between participants with high vs. low NT-proBNP levels as currently recommended for inclusion into clinical trials, which has important implications for future trial design.

## Conclusions

In HFpEF, circulating NT-proBNP levels exhibit prominent associations with proteins and pathways predominantly related to tissue fibrosis/ECM formation, inflammation, and angiogenesis. In particular, our findings suggest that higher NT-proBNP levels in HFpEF represent a marker of prominent fibrotic and inflammatory pathway activation in this patient population. Our findings advance our understanding of the clinical and biologic correlates of NT-proBNP in human HFpEF and will aid in the design and selection of participants in future HFpEF clinical trials, particularly those involving agents that act on these pathways.

## Supplementary Material

Supplementary material

## Figures and Tables

**Table 1:** Baseline characteristics of participants, across all 3 tertiles of SOMAScan-derived NT-proBNP in PHFS Numbers represent Mean (SD), Median (IQR) or counts (%).

Demographics	Lowest tertile *(n=84)*	Middle tertile *(n=85)*	Highest tertile (*n*=84)	*P* value
**SOMAScan NT-proBNP levels**	507-6558	6558-21474	21474-181192	
**Age, years**	53 (49.5-56.6)	59.1 (55.2-63)	63.3 (59.1-67.5)	0.0008
**Male Sex**	41 (48.81%)	49 (57.65%)	39 (46.43%)	0.3063
**Race**				0.3421
**White**	52 (61.9%)	65 (76.47%)	57 (67.46%)	
**Asian**	0 (0%)	1 (1.18%)	1 (1.19%)	
**Other**	4 (4.76%)	3 (3.53%)	4 (4.76%)	
**African American**	28 (33.33%)	16 (18.82%)	22 (26.19%)	
**Systolic BP, mmHg**	127 (122-132)	125 (121-130)	123 (118-128)	0.4605
**Diastolic BP, mmHg**	73.3 (70.7-75.8)	71.8 (69.2-74.3)	69 (66.5-71.4)	0.0520
**BMI, kg/m^2^**	32.8 (31-34.6)	33.6 (31.8-35.4)	27.9 (26.4-29.5)	<0.0001
**eGFR, mL/min/1.73m^2^**	61.9 (54.8-68.9)	50.8 (45.1-56.5)	39.9 (35.4-44.4)	<0.0001
**Diabetes**	25 (29.76%)	27 (31.76%)	23 (27.38%)	0.8228
**Stent**	8 (9.52%)	17 (20.00%)	23 (27.38%)	0.0123
**Bypass**	3 (3.57%)	10 (11.76%)	19 (22.62%)	0.0010
**Atrial fib/flutter**	14 (16.67%)	30 (35.29%)	41 (48.81%)	<0.0001
**Smoker**	7 (8.33%)	4 (4.71%)	4 (4.76%)	0.5211
**NYHA class**				0.0010
**NYHA 1**	27 (32.93%)	19 (22.62%)	4 (4.82%)	
**NYHA 2**	31 (37.80%)	39 (46.43%)	41 (49.40%)	
**NYHA 3**	20 (24.39%)	23 (27.38%)	34 (40.96%)	
**NYHA 4**	4 (4.88%)	3 (3.57%)	4 (4.82%)	
**Medication Use**				
**ACEI ARB**	59 (70.24%)	59 (69.41%)	36 (42.86%)	0.0002
**Aldosterone Antagonist**	7 (8.33%)	14 (16.47%)	13 (15.48%)	0.2401
**Aspirin**	42 (50.00%)	48 (56.47%)	45 (53.57%)	0.7001
**Beta Blocker**	52 (61.90%)	61 (71.76%)	59 (70.24%)	0.3361
**Calcium Channel Blocker**	25 (29.76%)	24 (28.24%)	23 (27.38%)	0.9417
**Hydralazine**	2 (2.38%)	4 (4.71%)	6 (7.14%)	0.3485
**Nitrate**	14 (16.67%)	8 (9.41%)	14 (16.67%)	0.2961
**Statin**	42 (50.00%)	44 (51.76%)	37 (44.05%)	0.5759
**Warfarin**	10 (11.90%)	22 (25.88%)	32 (38.10%)	0.0005
**Insulin**	11 (13.10%)	14 (16.47%)	8 (9.52%)	0.4070

ACEi/ARB= Angiotensin Converting Enzyme inhibitor/Angiotensin Receptor Blocker; Atrial fib/flutter=Atrial Fibrillation/Atrial Flutter; BMI=Body Mass Index; BP=Blood Pressure; eGFR= estimated Glomerular Filtration Rate; NYHA=New York Heart Association classification, NT-proBNP= N-terminal (NT)-pro hormone BNP.

**Table 2:** Baseline characteristics of participants, across all 3 tertiles of SOMAScan-derived NT-proBNP in TOPCAT Numbers represent Mean (SD), Median (IQR) or counts (%)

Demographics	Lowest tertile *(n=73)*	Middle tertile *(n=72)*	Highest tertile *(n=73)*	*P* value
**SOMAScan NT-proBNP levels**	773-8520	8520-15465	15465-60376	
**Age, years**	68 (60-75.3)	72 (66-79)	78 (66.8-82)	0.0003
**Male sex**	37 (50.68%)	44 (61.11%)	41 (56.16%)	0.4492
**Race**				0.4363
**White**	60 (82.19%)	61 (84.72%)	67 (91.78%)	
**Black**	12 (16.44%)	9 (12.50%)	5 (6.85%)	
**Asian**	0 (0.00%)	1 (1.39%)	0 (0.00%)	
**Other**	1 (1.37%)	1 (1.39%)	1 (1.37%)	
**Medical History**				
**Myocardial infarction**	18 (24.66%)	18 (25.00%)	13 (17.81%)	0.5027
**Stroke**	4 (5.48%)	5 (6.94%)	6 (8.22%)	0.8072
**CABG**	15 (20.55%)	20 (27.78%)	23 (31.51%)	0.3134
**PCI**	22 (30.14%)	18 (25.00%)	16 (21.92%)	0.5173
**COPD**	10 (13.70%)	8 (11.11%)	6 (8.22%)	0.5713
**Hypertension**	71 (97.26%)	64 (88.89%)	71 (97.26%)	0.0388
**AF**	22 (30.14%)	32 (44.44%)	54 (73.97%)	<0.0001
**Diabetes**	40 (54.79%)	30 (41.67%)	33 (45.21%)	0.2605
**Insulin use**	21 (28.77%)	16 (22.22%)	6 (8.22%)	0.0062
**Smoking**	37 (56.06%)	47 (69.12%)	43 (60.56%)	0.2849
**eGFR, mL/min/1.73m^2^**	66.3 (40-86.6)	64.7 (46.5-82.9)	63.4 (42.3-84.5)	0.6853
**Hematocrit, %**	38 (36-41.3)	39.4 (35.8-42)	39 (35.7-41.2)	0.6173
**BMI, kg/m^2^**	35.3 (30.7-41.1)	32.6 (28.8-35.5)	31.6 (27.2-35.4)	0.0012
**Systolic BP, mmHg**	126 (115-137)	122 (116-133)	124 (117-133)	0.8064
**Diastolic BP, mmHg**	70.2 (60-80.4)	70.4 (59.5-81.3)	67.7 (56.2-79.2)	0.2676
**Medication use**				
**Beta blocker**	58 (79.45%)	65 (90.28%)	58 (79.45%)	0.1346
**Calcium channel blocker**	30 (41.10%)	29 (40.28%)	26 (35.62%)	0.7651
**ACEI/ARB**	62 (84.93%)	44 (61.11%)	58 (79.45%)	0.0024
**Aspirin**	49 (67.12%)	48 (66.67%)	38 (52.05%)	0.1033
**Statin**	58 (79.45%)	51 (70.83%)	52 (71.23%)	0.4097
**KCCQ overall summary score**	62.5 (44.7-81.8)	70.3 (51.4-86.9)	59.5 (39.6-75.3)	0.0531
**KCCQ clinical summary score**	64.6 (45.8-82.6)	65.6 (44.5-84.6)	62.5 (45.8-77.6)	0.3133
**Participants with available NT-** **proBNP measurement in trial** *	15	26	30	
**Trial NT-proBNP level, ng/mL**	557 (356-757)	876 (645-1106)	1853 (1402-2303)	<0.0001
**Participants with available BNP** **measurement in trial** *	29	29	31	
**Trial BNP level, ng/mL**	180 (131-229)	211 (154-269)	477 (352-602)	<0.0001

ACEi/ARB= Angiotensin Converting Enzyme inhibitor/Angiotensin Receptor Blocker; AF=Atrial Fibrillation; BMI=Body Mass Index; CABG=Coronary Artery Bypass Graft; COPD=Chronic Obstructive Pulmonary Disease; BP=Blood Pressure; eGFR=estimated Glomerular Filtration Rate; KCCQ=Kansas City Cardiomyopathy Questionnaire, NT-proBNP= N-terminal (NT)-pro hormone BNP, PCI= Percutaneous Coronary Intervention.

* within 30 days prior to eligibility

## Novelty and Relevance

### What is New?

This is the first analysis investigating the relationship between plasma NT-proBNP and the plasma proteome in HFpEF. We used a broad proteomics platform of ~5000 plasma protein measurements, pathway analyses and concordance analysis in 2 well-characterized, independent HFpEF cohorts and identified novel associations between multiple plasma proteins and NT-proBNP, which indicate a relationship to pathways involved in ECM formation/fibrosis and inflammation. Our study advances our understanding of the clinical and biologic correlates of this widely used biomarker in HFpEF.

### Clinical Implications

Our study illustrates the stark differences in clinical and biological associations of high vs. low NT-proBNP levels in HFpEF. We show that elevated NT-proBNP levels in HFpEF serve as an indicator of significant activation of fibrotic and inflammatory pathways in this population. Our findings advance our interpretation of this widely used biomarker in HFpEF and will be facilitate the rational design of forthcoming HFpEF clinical trials, particularly those focusing on interventions targeting these specific pathways. Our findings can also aid in the interpretation of data from previous clinical trials.
