# Supplementary material for: Proteomic Associations of N-terminal (NT)-pro hormone BNP (NT-proBNP) in Heart Failure with Preserved Ejection Fraction (HFpEF)
Source: Circ Heart Fail. Author manuscript; Available in PMC 2024 Mar 1. (PMC7615693; doi:10.1161/CIRCHEARTFAILURE.123.011146)
Supplement: Supplementary material [file EMS194259-supplement-Supplementary_material.docx]

**SUPPLEMENTAL MATERIAL**

**Proteomic Associations of N-terminal (NT)-pro hormone BNP (NT-proBNP) in Heart Failure with Preserved Ejection Fraction (HFpEF)**

**Running title: Proteomics Associations of NT-proBNP in HFpEF**

Joe David Azzo, MD*^1^; Marie-Joe Dib, PhD*^2^; Loukas Zagkos, PhD^3^; Lei Zhao, MD, PhD^4^; Zhaoqing Wang, MS^4^; Ching-Pin Chang, MD, PhD ^4;^ Christina Ebert, PhD^4^; Oday Salman, MD^1^; Sushrima Gan, PhD^2^; Payman Zamani, MD, MTR^1,2^; Jordana B. Cohen, MD, MSCE^4,5^; Vanessa van Empel, MD, PhD^6^; A. Mark Richards, MD, PhD^7,8^; Ali Javaheri, MD, PhD^9,10^; Douglas L. Mann, MD^9^; Ernst Rietzschel, MD, PhD^11^; Peter Schafer, PhD^4^; Dietmar A. Seiffert, MD^4^; Dipender Gill, MD, PhD^3^; Stephen Burgess, PhD^12^; Francisco Ramirez-Valle, MD, PhD^4^; David A. Gordon, PhD^4^; Thomas P. Cappola, MD, ScM^1,2^; Julio A. Chirinos, MD, PhD^1,2^

*These authors contributed equally to this work.

^1^ University of Pennsylvania Perelman School of Medicine, Philadelphia, PA

^2^ Division of Cardiovascular Medicine, Hospital of the University of Pennsylvania, Philadelphia PA

^3^ Department of Epidemiology and Biostatistics, School of Public Health, Imperial College London, UK.

^4^ Bristol-Myers Squibb Company, Lawrenceville, NJ

^5^ Renal-Electrolyte and Hypertension Division, Perelman School of Medicine, University of Pennsylvania, Philadelphia PA

^6^ Department of Cardiology, Maastricht University Medical Center, Maastricht, The Netherlands

^7^ Cardiovascular Research Institute, National University of Singapore, Singapore.

^8^ Christchurch Heart Institute, University of Otago, Christchurch, New Zealand.

^9^ Washington University School of Medicine, St. Louis, MO.

^10^ John J. Cochran Veterans Hospital, St. Louis, MO.

^11^ Department of Cardiovascular Diseases, Ghent University Hospital, Ghent, Belgium

^12^ Department of Public Health and Primary Care, University of Cambridge, Cambridge, UK.

**Address for correspondence:**

Julio A. Chirinos, MD, PhD

South Tower, Rm. 11-138.

Perelman Center for Advanced Medicine.

3400 Civic Center Blvd.  Philadelphia, PA. 19104.

Tel: 215-573-6606; Fax: 215-746-7415

Email: julio.chirinos@uphs.upenn.edu

Twitter: @JulioChirinosMd

**Sensitivity analysis**

***Baseline clinical characteristics***

Participants with higher levels of NT-proBNP were more likely to be older, exhibited a lower BMI, lower estimated glomerular filtration rate (eGFR), a lower diastolic blood pressure, more advanced NYHA functional class, higher prevalence of atrial fibrillation, history of coronary revascularization, warfarin use and lower prevalence of ACE inhibitor and ARB use **(Table S6)**.

***Correlation to other proteins and biologic pathways***

In PHFS, we found 592 proteins to be significantly associated with the plasma levels of NT-proBNP in univariable analysis. A volcano plot showing the relationship between NT-proBNP and other plasma protein levels is shown in **Figure S6A.** Standard contrasts and P-values for the top 50 proteins are shows in **Table S7**.

The top 10 proteins associated with plasma levels of NT-proBNP were scavenger receptor class F member 2 (SCARF2, *Std Contrast* = 1.035567, *P*<0.0001), thrombospondin-2 (THBS2, *Std Contrast =* 1.064362*, P*<0.0001), angiopoietin-2 (ANGPT2, *Std Contrast =* 1.045554*, P*<0.0001), sushi, von Willebrand factor type A, EGF and pentraxin domain containing 1 (SVEP1, *Std Contrast* = 1.167521, *P*<0.0001), **EGF-containing Fibulin-like extracellular matrix protein 1** (EFEMP1, *Std Contrast* = 1.095649, *P*<0.0001), insulin-like growth factor binding protein 2 (IGFBP2, *Std Contrast* = 1.101989, *P*<0.0001), **Growth hormone receptor** (GHR, *Std Contrast* = -1.037458682, *P*<0.0001), **Follistatin-related protein 3** (FSTL3, *Std Contrast* = 1.009262, *P*<0.0001), **Growth arrest-specific protein 1 (GAS1,** *Std Contrast* = 1.031231, ***P*<0.0001) and Endothelial cell-specific molecule 1 (ESM1,** *Std Contrast* = 1.001607, ***P*<0.0001).**

After adjusting for sex, race, eGFR and additional covariates found to be significantly different across the two plasma NT-proBNP groups (age, history of stenting, bypass surgery, Atrial fibrillation, warfarin use, ACEi/ARB use and BMI, NYHA class, diastolic blood pressure) 240 proteins were found to be significantly different across levels (**Figure S6B**). Standard contrast and P-values for the top 50 proteins are shows in **Table S8**.

The top 10 proteins with a correlation to plasma levels of NT-pro BNP included, SVEP1 (*Std Contrast* = 0.923, *P*<0.0001), EFEMP1 (*Std Contrast* = 0.78, *P*<0.0001), peroxidasin homolog (PXDN*, Std Contrast* = 0.8, *P*<0.0001), **GAS1 (***Std Contrast* = 0.863, ***P*<0.0001), ESM1 (***Std Contrast* = 0.859, ***P*<0.0001),** THBS2 (*Std Contrast* = 0.871, *P*<0.0001), ANGPT2 (*Std Contrast* = 0.9, *P*<0.0001), golgi membrane protein 1 (GOLM1, *Std Contrast* = 0.983, *P*<0.0001), neuropilin-1 (NRP1, *Std Contrast* = 0.867, ***P*<0.0001)and pancreatic Ribonuclease (RNASE1,** *Std Contrast* = 0.78, ***P*<0.0001).**

The top canonical pathways significantly associated with NT-pro BNP in adjusted PHFS sensitivity analysis are shown **in Figure S4**. The top 5 signaling pathways that were significantly correlated with NT-proBNP included LXR/RXR activation pathway, hepatic fibrosis / hepatic stellate cell activation pathway, sperm motility pathway, IL-15 production pathway and inhibition of matrix metalloproteases pathway.

***Concordance Analysis***

A concordance plot in which the proteins that were positively or negatively related to NT-proBNP in the PHFS cohort in both the initial analysis and the sensitivity analysis is shown in **Figure S3**. We found 186 proteins that were concordantly associated with NT-proBNP. The top concordant proteins associated with NT-proBNP included SVEP-1, PXDN, ANGPT2 and Thrombospondin-2.

**Figure S1. Correlation between Somalogic NT-proBNP and immune-assay NT-proBNP in the PHFS HFpEF cohort among 68 participants exhibiting NT-proBNP levels in the relevant range (0-500 pg/ml). These data were fitted to obtain a linear model which estimated the SOMAScan® NT-proBNP cut-points corresponding to immunoassay cut-points of 360 pg/ml and 480 pg/ml, which were used in our sensitivity analyses**


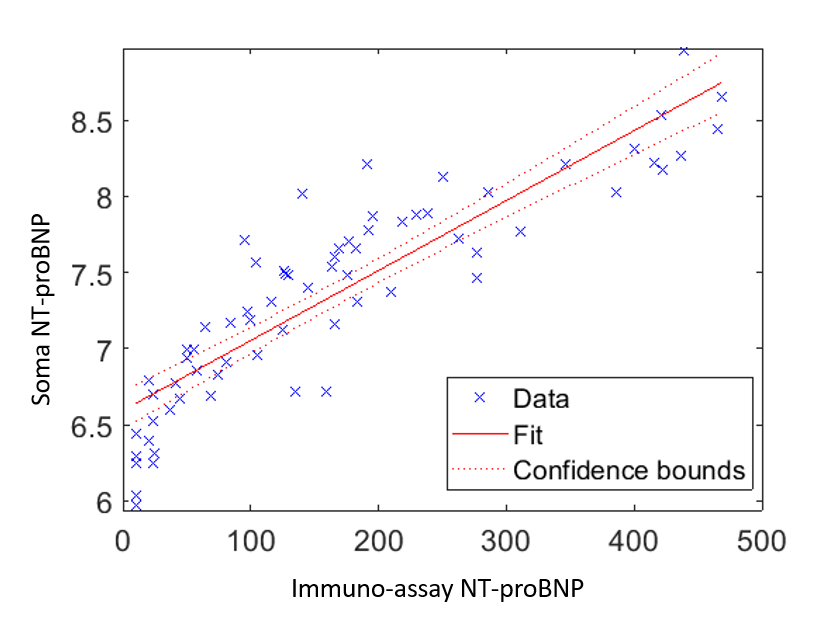


R=0.88; R^2^= 0.783

R=0.94 using the total range of immune-assay NT-proBNP

**Figure S2**. **Volcano plot demonstrating significant associations between all plasma proteins with plasma levels of N-terminal-pro BNP (NT-proBNP) in PHFS**


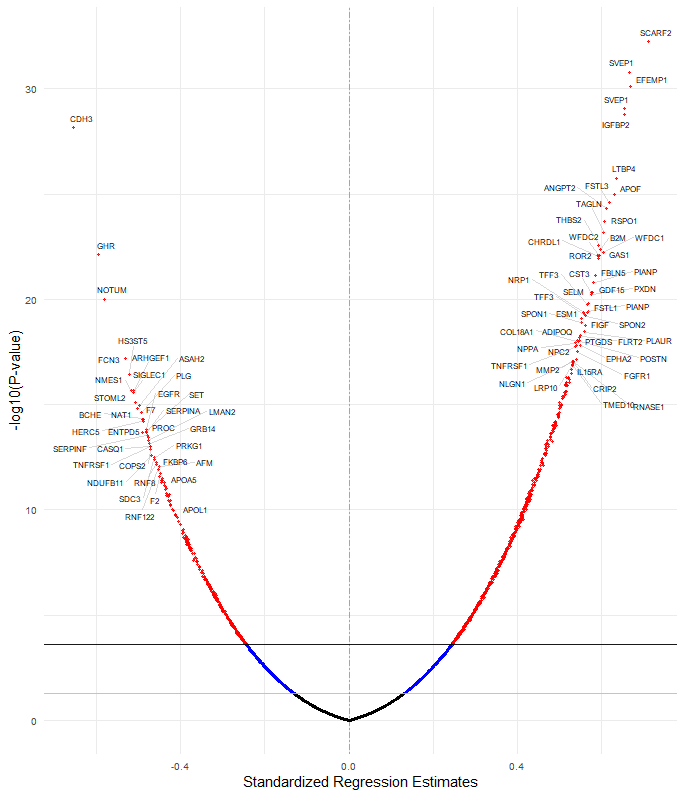


**Figure S3. Concordance between standardized beta estimate for proteome-wide association analyses in PHFS and standardized contrast in PHFS sensitivity analysis**

**
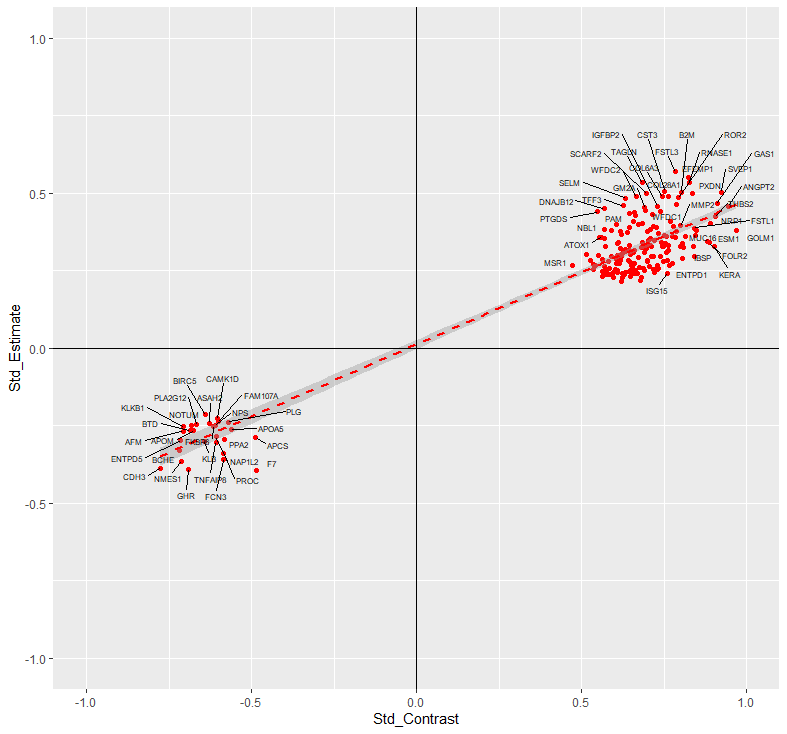
**

**Figure S4. Canonical pathway analysis of proteins observed to be significantly associated with plasma levels of NT-proBNP levels in the PHFS sensitivity analysis**

**Figure S5**. **Volcano plot demonstrating significant associations between all plasma proteins with plasma levels of N-terminal-pro BNP (NT-proBNP) in TOPCAT**


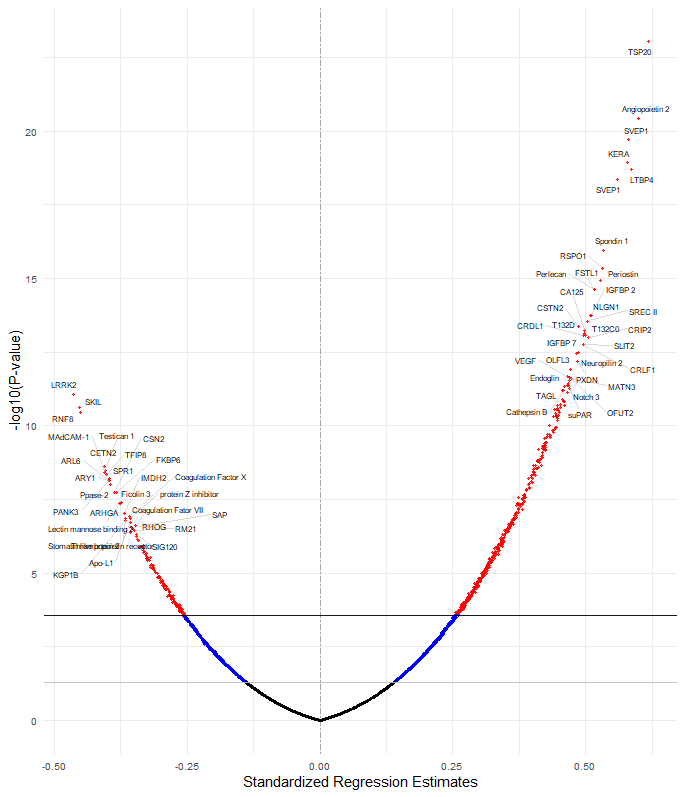


**Figure S6. Volcano plot demonstrating significant associations between all plasma proteins with plasma levels of N-terminal-pro BNP (NT-proBNP) in PHFS sensitivity analyses**

1. **PHFS univariable analysis**


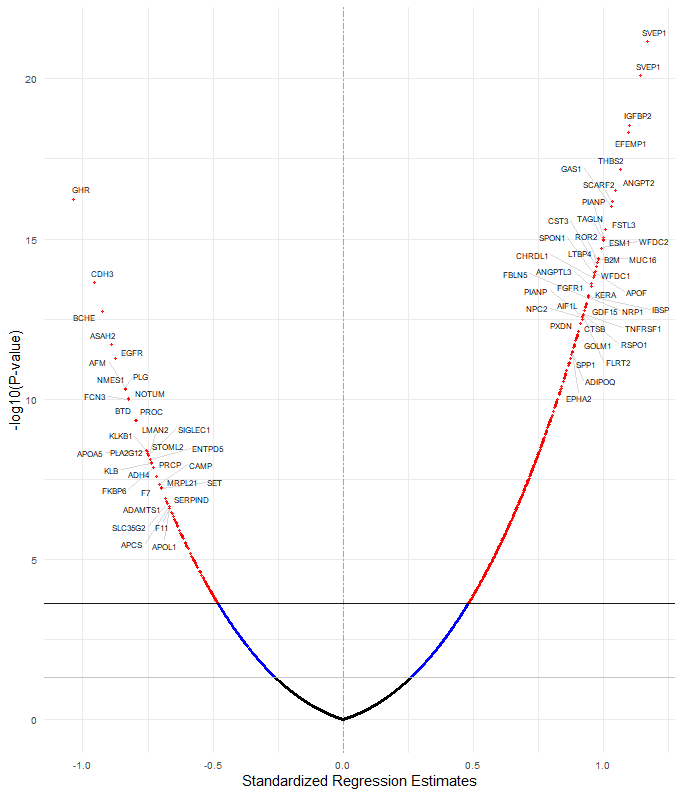


1. **PHFS adjusted**


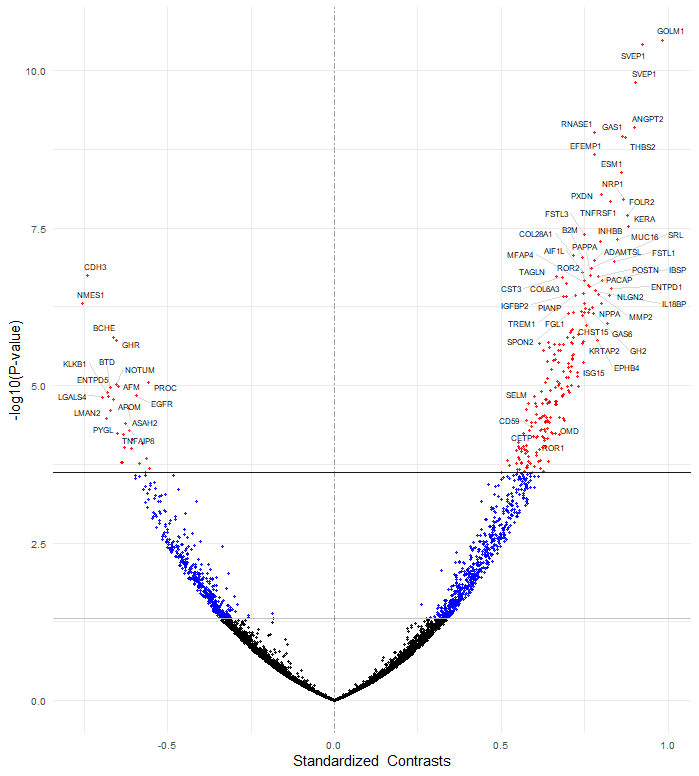


**Figure legends**

**Figure S1. Concordance between Somalogic NT-proBNP and immune-assay NT-proBNP in the PHFS HFpEF cohort**

**Figure S2**. **Volcano plot demonstrating significant associations between all plasma proteins with plasma levels of N-terminal-pro BNP (NT-proBNP) measured in the Penn Heart Failure Study (PHFS, n=253).** The plots show standardized beta estimates against the log-10 p value. The nominal and alpha-corrected significance levels are represented by solid lines on the y-axis.

**Figure S3. Correlation between Somalogic NT-proBNP and immune-assay NT-proBNP in the PHFS HFpEF cohort among 68 participants exhibiting NT-proBNP levels in the relevant range**

**Figure S4 Canonical pathway analysis of proteins observed to be significantly associated with plasma levels of NT-proBNP levels in the PHFS sensitivity analysis**. PCA corrected p-value of 0.05 threshold was used to determine significance. Numbers at the end of the bars indicate the Z-score corresponding to direction and strength of association.

**Figure S5**. **Volcano plot demonstrating significant associations between all plasma proteins with plasma levels of N-terminal-pro BNP (NT-proBNP) measured in the Treatment of Preserved Cardiac Function Heart Failure with an Aldosterone Antagonist Trial (TOPCAT, n=218).** The plots show standardized beta estimates against the log-10 p value. The nominal and alpha-corrected significance levels are represented by solid lines on the y-axis.

**Figure S6. Volcano plot demonstrating significant associations between all plasma proteins with plasma levels of N-terminal-pro BNP (NT-proBNP) measured in the Penn Heart Failure Study (PHFS, n=253) sensitivity analysis, in (A) univariable and (B) adjusted analyses.** The plots show standardized beta estimates against the log-10 p value. The nominal and alpha-corrected significance levels are represented by solid lines on the y-axis.

**Supplemental Tables**

**Table S1. Baseline characteristics of participants with and without available proteomics data in TOPCAT**

Numbers represent Mean (SD), Median (IQR) or counts (%).

| Demographics | Without Proteomics (n=3224) | With Proteomics  (n=218) | P-value |
| --- | --- | --- | --- |
| Age, years | 67.7 (67.3 to 68) | 71.2 (69.9 to 72.5) | <0.0001 |
| Male sex | 1546 (47.95%) | 122 (55.96%) | 0.0220 |
| Race |  |  | 0.4251 |
| White | 2871 (89.05%) | 188 (86.24%) |  |
| African American | 276 (8.56%) | 26 (11.93%) |  |
| Asian | 18 (0.56%) | 1 (0.46%) |  |
| Other | 67 (2.08%) | 3 (1.38%) |  |
| Medical History |  |  |  |
| Myocardial infarction | 844 (26.19%) | 49 (22.48%) | 0.2266 |
| Stroke | 250 (7.76%) | 15 (6.88%) | 0.6387 |
| CABG | 385 (11.95%) | 58 (26.61%) | <0.0001 |
| PCI | 444 (13.78%) | 56 (25.69%) | <0.0001 |
| COPD | 379 (11.76%) | 24 (11.01%) | 0.7389 |
| Hypertension | 2940 (91.22%) | 206 (94.50%) | 0.0945 |
| AF | 1105 (34.28%) | 108 (49.54%) | <0.0001 |
| Diabetes | 1015 (31.49%) | 103 (47.25%) | <0.0001 |
| Smoking | 1140 (39.65%) | 127 (61.95%) | <0.0001 |
| eGFR, mL/min/1.73m^2^ | 65.1 (64.4 to 65.7) | 61.7 (59.3 to 64.2) | 0.0100 |
| Hematocrit, % | 39.8 (39.7 to 40) | 38.6 (38 to 39.3) | 0.0008 |
| BMI, Kg/m^2^ | 31.2 (31 to 31.5) | 33.1 (32.2 to 34) | <0.0001 |
| Systolic BP, mmHg | 129 (128 to 129) | 124 (122 to 125) | <0.0001 |
| Diastolic BP, mmHg | 75.4 (75.1 to 75.8) | 68.5 (67.2 to 69.9) | <0.0001 |
| Insulin use | 384 (11.91%) | 43 (19.72%) | 0.0007 |
| Medications |  |  |  |
| Beta blocker | 2495 (77.41%) | 181 (83.03%) | 0.0536 |
| Calcium channel blocker | 1208 (37.48%) | 85 (38.99%) | 0.6559 |
| ACE ARB | 2735 (84.86%) | 164 (75.23%) | 0.0002 |
| Aspirin | 2115 (65.62%) | 135 (61.93%) | 0.2670 |
| Statin | 1644 (51.01%) | 161 (73.85%) | <0.0001 |
| KCCQ overall summary score | 49.7 (48.9 to 50.6) | 57.7 (53.9 to 61.4) | <0.0001 |
| KCCQ clinical summary score | 52.4 (51.5 to 53.2) | 57.9 (54.3 to 61.5) | 0.0016 |

ACEi/ARB= Angiotensin Converting Enzyme inhibitor/Angiotensin Receptor Blocker; AF=Atrial Fibrillation; BMI=Body Mass Index; CABG=Coronary Artery Bypass Graft; COPD=Chronic Obstructive Pulmonary Disease; BP=Blood Pressure; eGFR=estimated Glomerular Filtration Rate; KCCQ=Kansas City Cardiomyopathy Questionnaire, PCI= Percutaneous Coronary Intervention.

**Table S2. Top 50 proteins associated to NT-proBNP levels in the univariable analysis in PHFS**

| **Name** | **Uniprot ID** | **Std Estimate** | **Std CI95 LB** | **Std CI95 UB** | **P-value** | **FDR corrected**  **P-value** | **PCA corrected**  **P-value** |
| --- | --- | --- | --- | --- | --- | --- | --- |
| **SCARF2** | Q96GP6 | 0.712 | 0.611 | 0.813 | 5.56E-33 | 2.69E-28 | 1.84E-31 |
| **SVEP1** | Q4LDE5 | 0.666 | 0.569 | 0.763 | 1.66E-31 | 3.99E-27 | 3.44E-29 |
| **EFEMP1** | Q12805 | 0.669 | 0.57 | 0.768 | 8.16E-31 | 1.31E-26 | 1.62E-28 |
| **SVEP1** | Q4LDE5 | 0.655 | 0.555 | 0.754 | 8.91E-30 | 1.07E-25 | 7.43E-26 |
| **IGFBP2** | P18065 | 0.655 | 0.555 | 0.755 | 1.69E-29 | 1.63E-25 | 1.26E-24 |
| **LTBP4** | Q8N2S1 | 0.634 | 0.53 | 0.739 | 1.75E-26 | 1.20E-22 | 3.66E-24 |
| **APOF** | Q13790 | 0.63 | 0.525 | 0.736 | 1.06E-25 | 6.39E-22 | 2.22E-23 |
| **FSTL3** | O95633 | 0.618 | 0.514 | 0.723 | 2.53E-25 | 1.35E-21 | 5.22E-23 |
| **ANGPT2** | O15123 | 0.611 | 0.507 | 0.716 | 4.82E-25 | 2.32E-21 | 9.63E-23 |
| **RSPO1** | Q2MKA7 | 0.607 | 0.502 | 0.713 | 2.05E-24 | 8.99E-21 | 4.26E-22 |
| **TAGLN** | Q01995 | 0.604 | 0.498 | 0.711 | 7.01E-24 | 2.81E-20 | 1.46E-21 |
| **THBS2** | P35442 | 0.592 | 0.487 | 0.699 | 2.65E-23 | 9.84E-20 | 5.51E-21 |
| **B2M** | P61769 | 0.597 | 0.49 | 0.705 | 4.18E-23 | 1.44E-19 | 8.73E-21 |
| **WFDC1** | Q9HC57 | 0.603 | 0.494 | 0.712 | 5.86E-23 | 1.88E-19 | 1.22E-20 |
| **GAS1** | P54826 | 0.595 | 0.488 | 0.703 | 8.56E-23 | 2.20E-19 | 1.79E-20 |
| **WFDC2** | Q14508 | 0.593 | 0.485 | 0.7 | 8.59E-23 | 2.20E-19 | 1.79E-20 |
| **CHRDL1** | Q9BU40 | 0.59 | 0.483 | 0.697 | 8.67E-23 | 2.20E-19 | 1.80E-20 |
| **ROR2** | Q01974 | 0.593 | 0.485 | 0.701 | 1.16E-22 | 2.80E-19 | 2.42E-20 |
| **FBLN5** | Q9UBX5 | 0.585 | 0.476 | 0.694 | 7.26E-22 | 1.67E-18 | 1.51E-18 |
| **PIANP** | Q8IYJ0 | 0.581 | 0.472 | 0.69 | 1.58E-21 | 3.46E-18 | 3.30E-18 |
| **CST3** | P01034 | 0.575 | 0.465 | 0.685 | 4.73E-21 | 9.92E-18 | 9.87E-18 |
| **GDF15** | Q99988 | 0.577 | 0.467 | 0.687 | 4.95E-21 | 9.96E-18 | 1.03E-17 |
| **PXDN** | Q92626 | 0.575 | 0.466 | 0.686 | 5.99E-21 | 1.15E-17 | 1.24E-17 |
| **FSTL1** | Q12841 | 0.568 | 0.457 | 0.68 | 3.71E-20 | 6.17E-17 | 7.76E-17 |
| **SELM** | Q8WWX9 | 0.568 | 0.458 | 0.678 | 1.62E-20 | 2.90E-17 | 3.38E-16 |
| **TFF3** | Q07654 | 0.566 | 0.456 | 0.676 | 1.70E-20 | 2.93E-17 | 3.56E-16 |
| **PIANP** | Q8IYJ0 | 0.566 | 0.455 | 0.677 | 4.02E-20 | 6.46E-17 | 8.40E-16 |
| **NRP1** | O14786 | 0.557 | 0.448 | 0.666 | 4.15E-20 | 6.46E-17 | 8.66E-16 |
| **TFF3** | Q07654 | 0.558 | 0.448 | 0.668 | 5.44E-20 | 8.20E-17 | 1.14E-15 |
| **SPON2** | Q9BUD6 | 0.562 | 0.451 | 0.672 | 6.07E-20 | 8.87E-17 | 1.27E-15 |
| **ESM1** | Q9NQ30 | 0.551 | 0.442 | 0.66 | 8.01E-20 | 1.13E-16 | 1.67E-15 |
| **SPON1** | Q9HCB6 | 0.552 | 0.442 | 0.663 | 1.29E-19 | 1.77E-16 | 2.69E-15 |
| **FIGF** | O43915 | 0.561 | 0.449 | 0.674 | 1.80E-19 | 2.40E-16 | 3.76E-15 |
| **PTGDS** | P41222 | 0.546 | 0.435 | 0.658 | 6.23E-19 | 7.71E-16 | 5.20E-15 |
| **FLRT2** | O43155 | 0.55 | 0.438 | 0.662 | 5.50E-19 | 6.98E-16 | 5.73E-15 |
| **ADIPOQ** | Q15848 | 0.541 | 0.43 | 0.652 | 8.57E-19 | 1.03E-15 | 7.06E-15 |
| **PLAUR** | Q03405 | 0.559 | 0.446 | 0.673 | 3.53E-19 | 4.60E-16 | 7.38E-15 |
| **POSTN** | Q15063 | 0.548 | 0.435 | 0.66 | 9.30E-19 | 1.09E-15 | 7.75E-15 |
| **COL18A1** | P39060 | 0.544 | 0.432 | 0.656 | 1.02E-18 | 1.17E-15 | 8.84E-15 |
| **NPPA** | P01160 | 0.538 | 0.427 | 0.649 | 1.18E-18 | 1.32E-15 | 1.04E-14 |
| **EPHA2** | P29317 | 0.548 | 0.435 | 0.662 | 1.52E-18 | 1.65E-15 | 1.26E-14 |
| **NPC2** | P61916 | 0.539 | 0.428 | 0.651 | 1.55E-18 | 1.65E-15 | 1.29E-14 |
| **TNFRSF1** | Q9NS68 | 0.538 | 0.426 | 0.65 | 1.83E-18 | 1.92E-15 | 1.61E-14 |
| **FGFR1** | P11362 | 0.542 | 0.429 | 0.656 | 3.02E-18 | 3.10E-15 | 2.52E-14 |
| **IL15RA** | Q13261 | 0.539 | 0.425 | 0.653 | 7.27E-18 | 7.15E-15 | 6.03E-14 |
| **MMP2** | P08253 | 0.531 | 0.419 | 0.644 | 8.59E-18 | 8.25E-15 | 7.27E-14 |
| **RNASE1** | P07998 | 0.534 | 0.42 | 0.647 | 8.72E-18 | 8.25E-15 | 7.36E-14 |
| **CRIP2** | P52943 | 0.534 | 0.42 | 0.647 | 9.56E-18 | 8.77E-15 | 8.02E-14 |
| **NLGN1** | Q8N2Q7 | 0.53 | 0.418 | 0.643 | 9.63E-18 | 8.77E-15 | 8.63E-14 |
| **TMED10** | P49755 | 0.53 | 0.417 | 0.644 | 1.40E-17 | 1.23E-14 | 1.12E-13 |

**Table S3. Top 50 proteins associated to NT-proBNP levels in the adjusted analysis in PHFS**

| **Name** | **Uniprot ID** | **Std Estimate** | **Std CI95 LB** | **Std CI95 UB** | **P-value** | **FDR corrected**  **P-value** | **PCA corrected**  **P-value** |
| --- | --- | --- | --- | --- | --- | --- | --- |
| **SVEP1** | Q4LDE5 | 0.516 | 0.410 | 0.621 | 1.50E-18 | 7.27E-14 | 3.15E-16 |
| **SVEP1** | Q4LDE5 | 0.504 | 0.396 | 0.612 | 3.18E-17 | 6.98E-13 | 6.63E-15 |
| **EFEMP1** | Q12805 | 0.551 | 0.432 | 0.670 | 4.77E-17 | 6.98E-13 | 8.68E-15 |
| **FSTL3** | O95633 | 0.570 | 0.447 | 0.693 | 5.78E-17 | 6.98E-13 | 2.32E-14 |
| **ANGPT2** | O15123 | 0.459 | 0.354 | 0.564 | 1.53E-15 | 1.48E-11 | 3.24E-13 |
| **PXDN** | Q92626 | 0.499 | 0.383 | 0.616 | 4.45E-15 | 3.58E-11 | 9.28E-13 |
| **SCARF2** | Q96GP6 | 0.500 | 0.383 | 0.617 | 5.65E-15 | 3.89E-11 | 1.18E-12 |
| **B2M** | P61769 | 0.503 | 0.385 | 0.622 | 6.99E-15 | 4.22E-11 | 1.46E-12 |
| **RNASE1** | P07998 | 0.535 | 0.404 | 0.666 | 5.86E-14 | 3.14E-10 | 1.22E-11 |
| **WFDC1** | Q9HC57 | 0.463 | 0.348 | 0.578 | 9.51E-14 | 4.20E-10 | 1.98E-11 |
| **ROR2** | Q01974 | 0.487 | 0.367 | 0.608 | 9.56E-14 | 4.20E-10 | 2.01E-11 |
| **TNFRSF1** | Q9NS68 | 0.487 | 0.365 | 0.610 | 2.01E-13 | 7.83E-10 | 4.21E-11 |
| **TAGLN** | Q01995 | 0.535 | 0.400 | 0.670 | 2.13E-13 | 7.83E-10 | 4.45E-11 |
| **GAS1** | P54826 | 0.468 | 0.350 | 0.585 | 2.27E-13 | 7.83E-10 | 4.74E-11 |
| **CST3** | P01034 | 0.507 | 0.377 | 0.636 | 4.21E-13 | 1.35E-09 | 8.81E-11 |
| **IGFBP2** | P18065 | 0.458 | 0.341 | 0.576 | 5.32E-13 | 1.60E-09 | 1.11E-10 |
| **NRP1** | O14786 | 0.403 | 0.298 | 0.508 | 1.02E-12 | 2.92E-09 | 2.14E-10 |
| **SPON2** | Q9BUD6 | 0.459 | 0.338 | 0.580 | 1.62E-12 | 4.34E-09 | 3.38E-10 |
| **SELM** | Q8WWX9 | 0.484 | 0.356 | 0.611 | 1.90E-12 | 4.60E-09 | 3.98E-10 |
| **WFDC2** | Q14508 | 0.488 | 0.360 | 0.617 | 1.90E-12 | 4.60E-09 | 3.98E-10 |
| **RSPO1** | Q2MKA7 | 0.437 | 0.321 | 0.552 | 2.01E-12 | 4.60E-09 | 4.18E-10 |
| **THBS2** | P35442 | 0.426 | 0.314 | 0.539 | 2.18E-12 | 4.78E-09 | 4.55E-10 |
| **MMP2** | P08253 | 0.395 | 0.290 | 0.501 | 3.22E-12 | 6.77E-09 | 6.74E-10 |
| **LTBP4** | Q8N2S1 | 0.410 | 0.299 | 0.520 | 4.67E-12 | 9.40E-09 | 9.77E-10 |
| **TXNDC5** | Q8NBS9 | 0.441 | 0.322 | 0.560 | 5.26E-12 | 1.01E-08 | 1.10E-09 |
| **TIMP2** | P16035 | 0.394 | 0.287 | 0.500 | 5.59E-12 | 1.03E-08 | 1.16E-09 |
| **COL28A1** | Q2UY09 | 0.489 | 0.357 | 0.622 | 5.93E-12 | 1.05E-08 | 1.23E-09 |
| **FSTL1** | Q12841 | 0.388 | 0.283 | 0.493 | 6.11E-12 | 1.05E-08 | 1.27E-09 |
| **NPPA** | P01160 | 0.388 | 0.282 | 0.494 | 8.82E-12 | 1.46E-08 | 1.84E-09 |
| **PAM** | P19021 | 0.380 | 0.276 | 0.484 | 1.13E-11 | 1.83E-08 | 2.37E-09 |
| **LAMA2** | P07942 | 0.414 | 0.300 | 0.528 | 1.32E-11 | 1.94E-08 | 2.77E-09 |
| **LAMA2** | P11047 | 0.414 | 0.300 | 0.528 | 1.32E-11 | 1.94E-08 | 2.77E-09 |
| **LAMA2** | P24043 | 0.414 | 0.300 | 0.528 | 1.32E-11 | 1.94E-08 | 2.77E-09 |
| **POSTN** | Q15063 | 0.393 | 0.284 | 0.501 | 1.46E-11 | 2.08E-08 | 3.06E-09 |
| **TFF3** | Q07654 | 0.461 | 0.332 | 0.590 | 2.41E-11 | 3.32E-08 | 5.04E-09 |
| **COL6A3** | P12111 | 0.490 | 0.353 | 0.627 | 2.51E-11 | 3.37E-08 | 5.26E-09 |
| **DNAJB12** | Q9NXW2 | 0.450 | 0.323 | 0.576 | 3.05E-11 | 3.99E-08 | 6.39E-09 |
| **EPHA2** | P29317 | 0.431 | 0.309 | 0.554 | 3.87E-11 | 4.92E-08 | 8.09E-09 |
| **DKK3** | Q9UBP4 | 0.411 | 0.294 | 0.527 | 3.98E-11 | 4.93E-08 | 8.32E-09 |
| **CDH3** | P22223 | -0.386 | -0.496 | -0.277 | 4.51E-11 | 5.44E-08 | 9.43E-09 |
| **NLGN2** | Q8NFZ4 | 0.361 | 0.258 | 0.464 | 5.27E-11 | 6.20E-08 | 1.10E-08 |
| **TFF3** | Q07654 | 0.433 | 0.309 | 0.558 | 6.27E-11 | 7.21E-08 | 1.31E-08 |
| **FGFR1** | P11362 | 0.376 | 0.268 | 0.484 | 7.08E-11 | 7.95E-08 | 1.48E-08 |
| **MUC16** | Q8WXI7 | 0.380 | 0.271 | 0.490 | 8.11E-11 | 8.90E-08 | 1.69E-08 |
| **IGFLR1** | Q9H665 | 0.427 | 0.303 | 0.551 | 1.18E-10 | 1.27E-07 | 2.47E-08 |
| **PTGDS** | P41222 | 0.440 | 0.312 | 0.569 | 1.34E-10 | 1.41E-07 | 2.81E-08 |
| **GOLM1** | Q8NBJ4 | 0.380 | 0.268 | 0.491 | 1.70E-10 | 1.75E-07 | 3.57E-08 |
| **TMED10** | P49755 | 0.445 | 0.314 | 0.576 | 1.82E-10 | 1.83E-07 | 3.80E-08 |
| **TNFRSF1** | P19438 | 0.435 | 0.306 | 0.563 | 2.07E-10 | 2.02E-07 | 4.32E-08 |
| **UNC5B** | Q8IZJ1 | 0.413 | 0.291 | 0.535 | 2.09E-10 | 2.02E-07 | 4.37E-08 |

**Table S4. Top 50 proteins associated to NT-proBNP levels in the univariable analysis in TOPCAT**

| Name | Uniprot ID | Std Estimate | Std CI95 LB | Std CI95 UB | P-value | FDR corrected  P-value | PCA corrected  P-value |
| --- | --- | --- | --- | --- | --- | --- | --- |
| TSP20 | P35442 | 0.619 | 0.512 | 0.726 | 8.77E-24 | 1.41E-19 | 1.59E-21 |
| Angiopoietin 2 | O15123 | 0.598 | 0.486 | 0.710 | 3.68E-21 | 4.44E-17 | 6.71E-19 |
| SVEP1 | Q4LDE5 | 0.580 | 0.468 | 0.691 | 1.93E-20 | 1.86E-16 | 3.52E-18 |
| KERA | O60938 | 0.579 | 0.465 | 0.693 | 1.17E-19 | 9.44E-16 | 2.13E-17 |
| LTBP4 | Q8N2S1 | 0.587 | 0.470 | 0.703 | 1.96E-19 | 1.35E-15 | 3.59E-17 |
| SVEP1 | Q4LDE5 | 0.560 | 0.447 | 0.672 | 4.21E-19 | 2.54E-15 | 7.67E-17 |
| Spondin 1 | Q9HCB6 | 0.534 | 0.417 | 0.651 | 1.14E-16 | 6.12E-13 | 2.02E-14 |
| RSPO1 | Q2MKA7 | 0.532 | 0.413 | 0.651 | 4.46E-16 | 2.15E-12 | 8.08E-14 |
| Periostin | Q15063 | 0.527 | 0.407 | 0.647 | 1.16E-15 | 5.08E-12 | 2.02E-13 |
| FSTL1 | Q12841 | 0.516 | 0.397 | 0.635 | 2.30E-15 | 8.67E-12 | 4.24E-13 |
| Perlecan | P98160 | 0.516 | 0.397 | 0.635 | 2.33E-15 | 8.67E-12 | 4.24E-13 |
| IGFBP 2 | P18065 | 0.509 | 0.387 | 0.631 | 1.76E-14 | 5.97E-11 | 3.19E-12 |
| NLGN1 | Q8N2Q7 | 0.511 | 0.388 | 0.633 | 1.86E-14 | 5.97E-11 | 3.37E-12 |
| SREC II | Q96GP6 | 0.504 | 0.382 | 0.626 | 2.75E-14 | 8.31E-11 | 5.01E-12 |
| CSTN2 | Q9H4D0 | 0.486 | 0.368 | 0.605 | 4.15E-14 | 1.18E-10 | 7.56E-12 |
| CA125 | Q8WXI7 | 0.498 | 0.376 | 0.620 | 5.74E-14 | 1.54E-10 | 1.04E-11 |
| T132D | Q14C87 | 0.497 | 0.375 | 0.619 | 7.19E-14 | 1.82E-10 | 1.31E-11 |
| T132C0 | Q8N3T6 | 0.500 | 0.377 | 0.624 | 8.53E-14 | 1.98E-10 | 1.55E-11 |
| CRDL1 | Q9BU40 | 0.496 | 0.373 | 0.618 | 8.63E-14 | 1.98E-10 | 1.57E-11 |
| CRIP2 | P52943 | 0.505 | 0.380 | 0.630 | 1.03E-13 | 2.26E-10 | 1.87E-11 |
| SLIT2 | O94813 | 0.496 | 0.372 | 0.620 | 1.73E-13 | 3.59E-10 | 3.15E-11 |
| CRLF1 | O75462 | 0.496 | 0.372 | 0.620 | 1.79E-13 | 3.59E-10 | 3.25E-11 |
| Neuropilin 2 | O60462 | 0.486 | 0.362 | 0.609 | 3.26E-13 | 6.29E-10 | 5.93E-11 |
| IGFBP 7 | Q16270 | 0.482 | 0.359 | 0.604 | 3.56E-13 | 6.62E-10 | 6.48E-11 |
| PXDN | Q92626 | 0.484 | 0.360 | 0.609 | 6.60E-13 | 1.18E-09 | 1.20E-10 |
| OLFL3 | Q9NRN5 | 0.470 | 0.348 | 0.593 | 1.21E-12 | 2.08E-09 | 2.20E-10 |
| VEGF | O43915 | 0.466 | 0.343 | 0.590 | 2.13E-12 | 3.55E-09 | 3.88E-10 |
| MATN3 | O15232 | 0.469 | 0.345 | 0.593 | 2.32E-12 | 3.73E-09 | 4.22E-10 |
| OFUT2 | Q9Y2G5 | 0.474 | 0.348 | 0.599 | 2.48E-12 | 3.86E-09 | 4.51E-10 |
| Notch 3 | Q9UM47 | 0.469 | 0.344 | 0.594 | 2.79E-12 | 4.21E-09 | 5.07E-10 |
| Endoglin | P17813 | 0.468 | 0.343 | 0.592 | 3.51E-12 | 5.13E-09 | 6.38E-10 |
| MA1C1 | Q9NR34 | 0.467 | 0.342 | 0.593 | 4.16E-12 | 5.69E-09 | 7.57E-10 |
| CSMD2 | Q7Z408 | 0.465 | 0.340 | 0.590 | 4.22E-12 | 5.69E-09 | 7.69E-10 |
| EGFLA | Q63HQ2 | 0.466 | 0.341 | 0.592 | 4.24E-12 | 5.69E-09 | 7.72E-10 |
| TAGL | Q01995 | 0.460 | 0.336 | 0.583 | 4.73E-12 | 6.17E-09 | 8.61E-10 |
| suPAR | Q03405 | 0.469 | 0.343 | 0.596 | 5.48E-12 | 6.96E-09 | 9.97E-10 |
| Cathepsin B | P07858 | 0.459 | 0.334 | 0.583 | 6.20E-12 | 7.67E-09 | 1.13E-09 |
| BLC | O43927 | 0.456 | 0.332 | 0.579 | 6.51E-12 | 7.86E-09 | 1.18E-09 |
| TXD15 | Q96J42 | 0.465 | 0.339 | 0.592 | 7.40E-12 | 8.72E-09 | 1.35E-09 |
| LRRK2 | Q5S007 | -0.465 | -0.591 | -0.338 | 8.55E-12 | 9.84E-09 | 1.56E-09 |
| TIMP 2 | P16035 | 0.457 | 0.331 | 0.583 | 1.26E-11 | 1.41E-08 | 2.29E-09 |
| CHSTB | Q9NPF2 | 0.458 | 0.331 | 0.584 | 1.49E-11 | 1.61E-08 | 2.72E-09 |
| GPNMB0 | Q14956 | 0.460 | 0.333 | 0.587 | 1.49E-11 | 1.61E-08 | 2.72E-09 |
| SELM | Q8WWX9 | 0.453 | 0.327 | 0.579 | 1.90E-11 | 1.99E-08 | 3.45E-09 |
| SARP 2 | Q8N474 | 0.459 | 0.331 | 0.587 | 2.01E-11 | 2.06E-08 | 3.66E-09 |
| MIC-1 | Q99988 | 0.447 | 0.323 | 0.572 | 2.07E-11 | 2.08E-08 | 3.76E-09 |
| SKIL | P12757 | -0.453 | -0.579 | -0.326 | 2.33E-11 | 2.25E-08 | 4.24E-09 |
| Omentin | Q8WWA0 | 0.452 | 0.326 | 0.578 | 2.33E-11 | 2.25E-08 | 4.24E-09 |
| NLGN20 | Q8NFZ4 | 0.447 | 0.322 | 0.573 | 2.59E-11 | 2.45E-08 | 4.71E-09 |
| SMAC | Q9NR28 | 0.445 | 0.320 | 0.569 | 2.68E-11 | 2.49E-08 | 4.89E-09 |

**Table S5. Top 50 proteins associated to NT-proBNP levels in the adjusted analysis in TOPCAT**

| Name | Uniprot ID | Std Estimate | Std CI95 LB | Std CI95 UB | P-value | FDR corrected  P-value | PCA corrected  P-value |
| --- | --- | --- | --- | --- | --- | --- | --- |
| ANGPT2 | O15123 | 0.572 | 0.464 | 0.679 | 6.75E-21 | 3.26E-16 | 1.23E-18 |
| THBS2 | P35442 | 0.567 | 0.46 | 0.675 | 1.49E-20 | 3.60E-16 | 2.72E-18 |
| LTBP4 | Q8N2S1 | 0.526 | 0.419 | 0.633 | 1.48E-18 | 2.16E-14 | 2.71E-16 |
| PXDN | Q92626 | 0.585 | 0.466 | 0.704 | 1.79E-18 | 2.16E-14 | 3.26E-16 |
| SVEP1 | Q4LDE5 | 0.539 | 0.425 | 0.652 | 1.21E-17 | 1.10E-13 | 2.21E-15 |
| KERA | O60938 | 0.546 | 0.431 | 0.661 | 1.37E-17 | 1.10E-13 | 2.49E-15 |
| SVEP1 | Q4LDE5 | 0.517 | 0.404 | 0.63 | 1.58E-16 | 1.09E-12 | 2.02E-14 |
| TMEM132 | Q14C87 | 0.524 | 0.404 | 0.643 | 1.36E-15 | 8.23E-12 | 2.42E-13 |
| FSTL1 | Q12841 | 0.482 | 0.371 | 0.593 | 2.73E-15 | 1.46E-11 | 5.05E-13 |
| EGFLAM | Q63HQ2 | 0.483 | 0.372 | 0.595 | 3.48E-15 | 1.65E-11 | 6.26E-13 |
| HSPG2 | P98160 | 0.487 | 0.374 | 0.6 | 3.77E-15 | 1.65E-11 | 6.87E-13 |
| RSPO1 | Q2MKA7 | 0.53 | 0.406 | 0.654 | 5.31E-15 | 2.14E-11 | 9.69E-13 |
| NLGN1 | Q8N2Q7 | 0.498 | 0.381 | 0.616 | 7.99E-15 | 2.96E-11 | 1.45E-12 |
| CSMD2 | Q7Z408 | 0.481 | 0.367 | 0.596 | 1.28E-14 | 4.42E-11 | 2.34E-12 |
| OLFML3 | Q9NRN5 | 0.477 | 0.359 | 0.595 | 1.20E-13 | 3.88E-10 | 2.19E-11 |
| GAS1 | P54826 | 0.482 | 0.363 | 0.602 | 1.33E-13 | 4.04E-10 | 2.43E-11 |
| MAN1C1 | Q9NR34 | 0.476 | 0.356 | 0.596 | 2.56E-13 | 7.29E-10 | 4.67E-11 |
| SLIT2 | O94813 | 0.458 | 0.342 | 0.574 | 3.67E-13 | 9.84E-10 | 6.68E-11 |
| ROR2 | Q01974 | 0.501 | 0.372 | 0.629 | 6.26E-13 | 1.59E-09 | 1.14E-10 |
| CRIP2 | P52943 | 0.487 | 0.361 | 0.612 | 7.79E-13 | 1.84E-09 | 1.41E-10 |
| NRP2 | O60462 | 0.472 | 0.35 | 0.594 | 8.00E-13 | 1.84E-09 | 1.45E-10 |
| FAM163A | Q96GL9 | 0.453 | 0.336 | 0.57 | 8.76E-13 | 1.86E-09 | 1.59E-10 |
| SPON1 | Q9HCB6 | 0.464 | 0.344 | 0.584 | 8.87E-13 | 1.86E-09 | 1.61E-10 |
| FLT4 | P35916 | 0.46 | 0.341 | 0.579 | 1.09E-12 | 2.21E-09 | 2.01E-10 |
| MUC16 | Q8WXI7 | 0.451 | 0.333 | 0.569 | 1.53E-12 | 2.96E-09 | 2.79E-10 |
| SELM | Q8WWX9 | 0.494 | 0.365 | 0.624 | 1.59E-12 | 2.96E-09 | 2.90E-10 |
| GAS6 | Q14393 | 0.448 | 0.331 | 0.566 | 1.72E-12 | 3.08E-09 | 3.14E-10 |
| SPON2 | Q9BUD6 | 0.446 | 0.328 | 0.563 | 1.92E-12 | 3.31E-09 | 3.49E-10 |
| CELA1 | Q9UNI1 | 0.438 | 0.322 | 0.554 | 2.77E-12 | 4.62E-09 | 5.05E-10 |
| TMEM132 | Q8N3T6 | 0.45 | 0.33 | 0.57 | 3.36E-12 | 5.24E-09 | 6.11E-10 |
| FSTL3 | O95633 | 0.483 | 0.354 | 0.612 | 3.45E-12 | 5.24E-09 | 6.28E-10 |
| ENG | P17813 | 0.437 | 0.321 | 0.554 | 3.47E-12 | 5.24E-09 | 6.32E-10 |
| POFUT2 | Q9Y2G5 | 0.433 | 0.316 | 0.549 | 5.09E-12 | 7.45E-09 | 9.27E-10 |
| WFDC1 | Q9HC57 | 0.464 | 0.339 | 0.589 | 6.52E-12 | 9.27E-09 | 1.18E-09 |
| TIMP1 | P01033 | 0.425 | 0.31 | 0.54 | 7.30E-12 | 1.01E-08 | 1.33E-09 |
| FIGF | O43915 | 0.443 | 0.323 | 0.564 | 7.73E-12 | 1.03E-08 | 1.40E-09 |
| FLRT2 | O43155 | 0.429 | 0.312 | 0.546 | 8.07E-12 | 1.03E-08 | 1.46E-09 |
| IGSF8 | Q969P0 | 0.435 | 0.317 | 0.554 | 8.16E-12 | 1.03E-08 | 1.48E-09 |
| TIMP2 | P16035 | 0.431 | 0.314 | 0.549 | 8.43E-12 | 1.04E-08 | 1.53E-09 |
| ADAM11 | O75078 | 0.439 | 0.319 | 0.559 | 9.62E-12 | 1.16E-08 | 1.75E-09 |
| SFTPC | P11686 | 0.436 | 0.317 | 0.555 | 1.01E-11 | 1.17E-08 | 1.82E-09 |
| LIFR | P42702 | 0.428 | 0.31 | 0.545 | 1.28E-11 | 1.47E-08 | 2.33E-09 |
| NLGN2 | Q8NFZ4 | 0.431 | 0.312 | 0.549 | 1.37E-11 | 1.53E-08 | 2.49E-09 |
| LRP10 | Q7Z4F1 | 0.444 | 0.321 | 0.566 | 1.48E-11 | 1.62E-08 | 2.69E-09 |
| CHST11 | Q9NPF2 | 0.424 | 0.306 | 0.542 | 2.11E-11 | 2.25E-08 | 3.82E-09 |
| IGFBP2 | P18065 | 0.464 | 0.335 | 0.594 | 2.23E-11 | 2.35E-08 | 4.07E-09 |
| CD93 | Q9NPY3 | 0.432 | 0.312 | 0.553 | 2.29E-11 | 2.35E-08 | 4.17E-09 |
| RNF8 | O76064 | -0.423 | -0.541 | -0.304 | 2.93E-11 | 2.95E-08 | 5.34E-09 |
| GDF15 | Q99988 | 0.438 | 0.314 | 0.561 | 3.61E-11 | 3.56E-08 | 6.57E-09 |
| PLA2R1 | Q13018 | 0.413 | 0.296 | 0.529 | 3.90E-11 | 3.77E-08 | 7.10E-09 |

**Table S6.** **Baseline characteristics of participants in PHFS sensitivity analysis**

Numbers represent Mean (SD), Median (IQR) or counts (%).

| Demographics | NT-proBNP below threshold  (*n*=166) | NT-proBNP above threshold  (*n*=87) | P-value |
| --- | --- | --- | --- |
| Age, years | 56.1 (53.4 to 58.7) | 62.9 (58.7 to 67) | 0.0050 |
| Male sex | 88 (53.01%) | 41 (47.13%) | 0.3737 |
| Race |  |  | 0.9153 |
| White | 114 (68.67%) | 60 (68.97%) |  |
| Asian | 1 (0.60%) | 1 (1.15%) |  |
| Other | 7 (4.22%) | 4 (4.60%) |  |
| African American | 44 (26.51%) | 22 (25.29%) |  |
| Systolic BP, mmHg | 127 (123 to 130) | 122 (118 to 127) | 0.1280 |
| Diastolic BP. mmHg | 72.8 (70.9 to 74.6) | 68.6 (66.2 to 71) | 0.0066 |
| BMI, Kg/m^2^ | 33 (31.7 to 34.3) | 28.4 (26.8 to 29.9) | <0.0001 |
| eGFR, mL/min/1.73m2 | 56.7 (52.2 to 61.2) | 39.5 (35.2 to 43.9) | <0.0001 |
| Diabetes | 50 (30.12%) | 25 (28.74%) | 0.8188 |
| Stent | 24 (14.46%) | 24 (27.59%) | 0.0114 |
| Bypass | 11 (6.63%) | 21 (24.14%) | <0.0001 |
| Atrial fib flutter | 46 (27.71%) | 39 (44.83%) | 0.0062 |
| Smoker | 11 (6.63%) | 4 (4.60%) | 0.5163 |
| NYHA class |  |  | 0.0008 |
| NHYA 1 | 45 (27.61%) | 5 (5.81%) |  |
| NYHA 2 | 69 (42.33%) | 42 (48.84%) |  |
| NYHA 3 | 41 (25.15%) | 36 (41.86%) |  |
| NYHA 4 | 8 (4.91%) | 3 (3.49%) |  |
| Medication use |  |  |  |
| ACEI ARB | 113 (68.07%) | 41 (47.13%) | 0.0012 |
| Aldosterone Antagonist | 18 (10.84%) | 16 (18.39%) | 0.0945 |
| Aspirin | 86 (51.81%) | 49 (56.32%) | 0.4941 |
| Beta Blocker | 110 (66.27%) | 62 (71.26%) | 0.4182 |
| Calcium Channel Blocker | 49 (29.52%) | 23 (26.44%) | 0.6059 |
| Hydralazine | 5 (3.01%) | 7 (8.05%) | 0.1154 |
| Nitrate | 22 (13.25%) | 14 (16.09%) | 0.5392 |
| Statin | 81 (48.80%) | 42 (48.28%) | 0.9374 |
| Warfarin | 34 (20.48%) | 30 (34.48%) | 0.0150 |
| Insulin | 25 (15.06%) | 8 (9.20%) | 0.1883 |

ACEi/ARB= Angiotensin Converting Enzyme inhibitor/Angiotensin Receptor Blocker; Atrial fib/flutter=Atrial Fibrillation/Atrial Flutter; BMI=Body Mass Index; DBP=Diastolic Blood Pressure; eGFR= estimated Glomerular Filtration Rate; NYHA=New York Heart Association classification; SBP=Systolic Blood Pressure

**Table S7. Top 50 proteins associated to NT-proBNP levels in the univariable analysis in PHFS sensitivity analysis**

| **Name** | **Uniprot ID** | **P-value** | **PCA corrected**  **P-value** | **FDR corrected**  **P-value** | **Standardized Contrast** |
| --- | --- | --- | --- | --- | --- |
| **SVEP1** | Q4LDE5 | 6.69E-22 | 1.29E-19 | 3.23E-17 | 1.168 |
| **SVEP1** | Q4LDE5 | 7.80E-21 | 1.51E-18 | 1.88E-16 | 1.142 |
| **IGFBP2** | P18065 | 2.77E-19 | 4.63E-17 | 4.47E-15 | 1.102 |
| **EFEMP1** | Q12805 | 4.82E-19 | 8.48E-17 | 5.81E-15 | 1.096 |
| **THBS2** | P35442 | 6.75E-18 | 1.16E-15 | 6.51E-14 | 1.064 |
| **ANGPT2** | O15123 | 3.10E-17 | 5.48E-15 | 2.49E-13 | 1.046 |
| **GHR** | P10912 | 5.90E-17 | 2.32E-14 | 4.07E-13 | -1.037 |
| **GAS1** | P54826 | 9.63E-17 | 2.32E-14 | 5.16E-13 | 1.031 |
| **SCARF2** | Q96GP6 | 6.85E-17 | 2.32E-14 | 4.13E-13 | 1.036 |
| **FSTL3** | O95633 | 5.21E-16 | 1.16E-13 | 2.51E-12 | 1.009 |
| **ESM1** | Q9NQ30 | 9.26E-16 | 1.85E-13 | 4.01E-12 | 1.002 |
| **PIANP** | Q8IYJ0 | 1.01E-15 | 2.08E-13 | 4.01E-12 | 1 |
| **TAGLN** | Q01995 | 1.07E-15 | 2.32E-13 | 4.01E-12 | 1 |
| **WFDC2** | Q14508 | 1.92E-15 | 3.94E-13 | 6.62E-12 | 0.992 |
| **B2M** | P61769 | 4.10E-15 | 8.58E-13 | 1.32E-11 | 0.981 |
| **MUC16** | Q8WXI7 | 4.45E-15 | 9.28E-13 | 1.34E-11 | 0.98 |
| **ROR2** | Q01974 | 5.24E-15 | 1.09E-12 | 1.48E-11 | 0.978 |
| **CST3** | P01034 | 7.27E-15 | 1.53E-12 | 1.89E-11 | 0.973 |
| **LTBP4** | Q8N2S1 | 7.44E-15 | 1.55E-12 | 1.89E-11 | 0.973 |
| **WFDC1** | Q9HC57 | 1.05E-14 | 2.20E-12 | 2.54E-11 | 0.968 |
| **APOF** | Q13790 | 1.11E-14 | 2.32E-12 | 2.54E-11 | 0.968 |
| **SPON1** | Q9HCB6 | 1.33E-14 | 2.80E-12 | 2.93E-11 | 0.965 |
| **CHRDL1** | Q9BU40 | 1.61E-14 | 3.38E-12 | 3.39E-11 | 0.962 |
| **CDH3** | P22223 | 2.35E-14 | 4.91E-12 | 4.73E-11 | -0.957 |
| **KERA** | O60938 | 2.48E-14 | 5.19E-12 | 4.79E-11 | 0.956 |
| **ANGPTL3** | Q9Y5C1 | 2.98E-14 | 6.24E-12 | 5.50E-11 | 0.953 |
| **FGFR1** | P11362 | 5.60E-14 | 1.17E-11 | 1.01E-10 | 0.944 |
| **IBSP** | P21815 | 6.26E-14 | 1.31E-11 | 1.04E-10 | 0.943 |
| **NRP1** | O14786 | 6.29E-14 | 1.31E-11 | 1.04E-10 | 0.942 |
| **FBLN5** | Q9UBX5 | 6.83E-14 | 1.42E-11 | 1.09E-10 | 0.941 |
| **HS6ST2** | Q96MM7 | 1.00E-13 | 2.09E-11 | 1.55E-10 | 0.936 |
| **GDF15** | Q99988 | 1.07E-13 | 2.24E-11 | 1.62E-10 | 0.935 |
| **RNASE1** | P07998 | 1.28E-13 | 2.68E-11 | 1.88E-10 | 0.932 |
| **FSTL1** | Q12841 | 1.36E-13 | 2.85E-11 | 1.94E-10 | 0.931 |
| **CLSTN2** | Q9H4D0 | 1.69E-13 | 3.53E-11 | 2.33E-10 | 0.928 |
| **BCHE** | P06276 | 1.81E-13 | 3.78E-11 | 2.42E-10 | -0.927 |
| **TNFRSF1** | Q9NS68 | 2.20E-13 | 4.59E-11 | 2.87E-10 | 0.924 |
| **RSPO1** | Q2MKA7 | 2.26E-13 | 4.73E-11 | 2.88E-10 | 0.923 |
| **CTSB** | P07858 | 2.34E-13 | 4.90E-11 | 2.90E-10 | 0.923 |
| **FLRT2** | O43155 | 2.46E-13 | 5.16E-11 | 2.98E-10 | 0.922 |
| **AIF1L** | Q9BQI0 | 2.61E-13 | 5.45E-11 | 3.05E-10 | 0.921 |
| **PIANP** | Q8IYJ0 | 2.65E-13 | 5.55E-11 | 3.05E-10 | 0.921 |
| **NPC2** | P61916 | 2.81E-13 | 5.88E-11 | 3.16E-10 | 0.92 |
| **PXDN** | Q92626 | 3.15E-13 | 6.60E-11 | 3.46E-10 | 0.918 |
| **MMP2** | P08253 | 4.24E-13 | 8.86E-11 | 4.55E-10 | 0.914 |
| **MFAP4** | P55083 | 7.44E-13 | 1.55E-10 | 7.81E-10 | 0.905 |
| **TFF3** | Q07654 | 7.76E-13 | 1.62E-10 | 7.97E-10 | 0.904 |
| **GOLM1** | Q8NBJ4 | 9.48E-13 | 1.98E-10 | 9.53E-10 | 0.901 |
| **COL28A1** | Q2UY09 | 1.02E-12 | 2.14E-10 | 1.01E-09 | 0.9 |
| **ADAMTSL** | Q86TH1 | 1.07E-12 | 2.24E-10 | 1.03E-09 | 0.899 |

**Table S8. Top 50 proteins associated to NT-proBNP levels in the adjusted analysis in PHFS sensitivity analysis**

| Name | Uniprot ID | P-value | PCA corrected  P-value | FDR corrected  P-value | Standardized contrast |
| --- | --- | --- | --- | --- | --- |
| GOLM1 | Q8NBJ4 | 3.30E-11 | 6.90E-09 | 9.30E-07 | 0.983 |
| SVEP1 | Q4LDE5 | 3.85E-11 | 8.05E-09 | 9.30E-07 | 0.923 |
| SVEP1 | Q4LDE5 | 1.55E-10 | 3.25E-08 | 2.50E-06 | 0.901 |
| ANGPT2 | O15123 | 8.11E-10 | 1.70E-07 | 7.86E-06 | 0.900 |
| RNASE1 | P07998 | 9.54E-10 | 1.99E-07 | 7.86E-06 | 0.780 |
| GAS1 | P54826 | 1.09E-09 | 2.29E-07 | 7.86E-06 | 0.863 |
| THBS2 | P35442 | 1.13E-09 | 2.38E-07 | 7.86E-06 | 0.871 |
| EFEMP1 | Q12805 | 2.14E-09 | 4.49E-07 | 1.30E-05 | 0.780 |
| ESM1 | Q9NQ30 | 4.19E-09 | 8.77E-07 | 2.25E-05 | 0.859 |
| PXDN | Q92626 | 9.12E-09 | 1.91E-06 | 4.40E-05 | 0.800 |
| NRP1 | O14786 | 1.09E-08 | 2.29E-06 | 4.81E-05 | 0.867 |
| TNFRSF1 | Q9NS68 | 1.21E-08 | 2.54E-06 | 4.89E-05 | 0.828 |
| FOLR2 | P14207 | 1.97E-08 | 4.13E-06 | 7.34E-05 | 0.879 |
| KERA | O60938 | 2.93E-08 | 6.13E-06 | 1.01E-04 | 0.881 |
| FSTL3 | O95633 | 3.99E-08 | 8.35E-06 | 1.29E-04 | 0.748 |
| MUC16 | Q8WXI7 | 4.80E-08 | 1.00E-05 | 1.45E-04 | 0.849 |
| INHBB | P09529 | 5.11E-08 | 1.07E-05 | 1.45E-04 | 0.799 |
| COL28A1 | Q2UY09 | 8.55E-08 | 1.79E-05 | 2.29E-04 | 0.717 |
| B2M | P61769 | 9.15E-08 | 1.91E-05 | 2.33E-04 | 0.742 |
| ADAMTSL | Q86TH1 | 1.02E-07 | 2.15E-05 | 2.45E-04 | 0.779 |
| FSTL1 | Q12841 | 1.06E-07 | 2.23E-05 | 2.45E-04 | 0.839 |
| SRL | Q86TD4 | 1.38E-07 | 2.90E-05 | 3.04E-04 | 0.769 |
| AIF1L | Q9BQI0 | 1.57E-07 | 3.30E-05 | 3.29E-04 | 0.744 |
| PAPPA | Q13219 | 1.77E-07 | 3.72E-05 | 3.29E-04 | 0.766 |
| CDH3 | P22223 | 1.80E-07 | 3.78E-05 | 3.29E-04 | -0.741 |
| TAGLN | Q01995 | 1.82E-07 | 3.82E-05 | 3.29E-04 | 0.667 |
| POSTN | Q15063 | 1.88E-07 | 3.95E-05 | 3.29E-04 | 0.791 |
| CST3 | P01034 | 1.90E-07 | 3.99E-05 | 3.29E-04 | 0.682 |
| IBSP | P21815 | 2.13E-07 | 4.46E-05 | 3.47E-04 | 0.804 |
| ROR2 | Q01974 | 2.15E-07 | 4.51E-05 | 3.47E-04 | 0.750 |
| COL6A3 | P12111 | 2.43E-07 | 5.09E-05 | 3.79E-04 | 0.694 |
| MFAP4 | P55083 | 2.55E-07 | 5.34E-05 | 3.86E-04 | 0.761 |
| PACAP | Q8WU39 | 2.72E-07 | 5.69E-05 | 3.98E-04 | 0.765 |
| ENTPD1 | P49961 | 2.91E-07 | 6.08E-05 | 4.13E-04 | 0.829 |
| IL18BP | O95998 | 3.14E-07 | 6.58E-05 | 4.34E-04 | 0.781 |
| WFDC1 | Q9HC57 | 3.47E-07 | 7.26E-05 | 4.60E-04 | 0.745 |
| MMP2 | P08253 | 3.64E-07 | 7.61E-05 | 4.60E-04 | 0.792 |
| TREM1 | Q9NP99 | 3.65E-07 | 7.64E-05 | 4.60E-04 | 0.724 |
| NLGN2 | Q8NFZ4 | 3.77E-07 | 7.90E-05 | 4.60E-04 | 0.823 |
| IGFBP2 | P18065 | 3.81E-07 | 7.98E-05 | 4.60E-04 | 0.685 |
| PIANP | Q8IYJ0 | 3.90E-07 | 8.16E-05 | 4.60E-04 | 0.696 |
| NPPA | P01160 | 4.90E-07 | 1.03E-04 | 5.50E-04 | 0.800 |
| LRP10 | Q7Z4F1 | 4.94E-07 | 1.03E-04 | 5.50E-04 | 0.748 |
| NMES1 | Q9C002 | 5.01E-07 | 1.05E-04 | 5.50E-04 | -0.755 |
| HSPG2 | P98160 | 5.86E-07 | 1.23E-04 | 6.29E-04 | 0.773 |
| FLRT2 | O43155 | 6.02E-07 | 1.26E-04 | 6.32E-04 | 0.753 |
| GUCA2B | Q16661 | 6.21E-07 | 1.30E-04 | 6.39E-04 | 0.763 |
| LTBP4 | Q8N2S1 | 6.68E-07 | 1.40E-04 | 6.57E-04 | 0.740 |
| CLSTN2 | Q9H4D0 | 6.79E-07 | 1.42E-04 | 6.57E-04 | 0.750 |
| FGFR1 | P11362 | 6.93E-07 | 1.45E-04 | 6.57E-04 | 0.762 |
